# STAT3 determines IL-4 signalling outcomes in naïve T cells

**DOI:** 10.1038/s41598-021-89860-7

**Published:** 2021-05-18

**Authors:** Lachlan P. Deimel, Zheyi Li, Sreeja Roy, Charani Ranasinghe

**Affiliations:** 1 Molecular Mucosal Vaccine Immunology Group, Department of Immunology and Infectious Disease, The John Curtin School of Medical Research, The Australian National University, Canberra, ACT 2601 Australia; 2 Sir William Dunn School of Pathology, Medical Sciences Division, University of Oxford, Oxfordshire, OX1 3RE UK; 3 Department of Immunology and Microbial Disease, Albany Medical College, Albany, NY 12208 USA

**Keywords:** Immunology, Adaptive immunity, Cytokines, Immune evasion, Infection, Vaccines

## Abstract

IL-4 production is associated with low-avidity, poorly cytotoxic T cell induction that contributes to viral immune evasion and the failure of T cell-based vaccines. Yet, the precise mechanisms that regulate IL-4 signalling in T cells remain elusive. Mounting evidence indicates that cells can dynamically alter their IL-4/IL-13 receptor signature to modulate downstream immune outcomes upon pathogen encounter. Here, we describe how naïve (CD62L^+^CD44^lo–mid^) CD4 and CD8 T cells distinctly engage both STAT6 and STAT3 in response to IL-4. We further show that IL-4R⍺ expression is both time- and IL-4 concentration-dependent. Remarkably, our findings reveal that STAT3 inhibition can ablate IL-4R⍺ and affect transcriptional expression of other *Stat* and *Jak* family members. By extension, the loss of STAT3 lead to aberrant STAT6 phosphorylation, revealing an inter-regulatory relationship between the two transcription factors. Moreover, IL-4 stimulation down-regulated TGF-β1 and IFN-γR1 expression on naïve T cells, possibly signifying the broad regulatory implications of IL-4 in conditioning lineage commitment decisions during early infection. Surprisingly, naïve T cells were unresponsive to IL-13 stimulation, unlike dendritic cells. Collectively, these findings could be exploited to inform more efficacious vaccines, as well as design treatments against IL-4/IL-13-associated disease conditions.

## Introduction

Since the discovery of interleukin (IL)-4, its multifunctional implications in mediating adaptive immunity have been well-studied^1,2^. In particular, IL-4 promotes humoral responses whilst dampening cell-mediated activity, including via type 2 helper T cell lineage commitment and maintenance^1,3,4^. Consequently, its production and signalling has been implicated in many different pathologies, including infection, cancer, autoimmunity, immunodeficiency and allergy^5–7^.

Studies have also shown the importance of IL-4 in determining vaccination outcomes. Kienzle et *al*. were among the first to demonstrate that IL-4 availability promotes a noncytolytic CD8^low^ immune phenotype^8^. Interestingly, some viruses (such as HIV) exploit IL-4 production to evade cytotoxic T cell-mediated elimination^9^. Indeed, endogenous IL-4 production serves as an attractive explanation as to why cytotoxic T cell-based approaches to HIV-1 vaccination design have thus far yielded limited protective efficacy^10,11^. Following decades of disappointing outcomes with many HIV-1 vaccine candidates, including the recent HVTN 701 trial^12^, as well as the ongoing SARS-CoV2 pandemic, nuanced approaches to vaccination design is of great importance. Studies in our laboratory have shown that a pox viral vector-based prime-boost vaccine strategy that co-expresses HIV antigens together with an IL-4 receptor antagonist can drastically improve avidity, poly-functionality and cytotoxicity of mucosal and systemic HIV-specific CD4 and CD8 T cells, associated with protection in both mice and macaques^10,13–15^. While this is a hopeful step towards establishing protective immunity against HIV-1, how different T cells govern and regulate their response to different IL-4 conditions at the molecular level still remains unclear.

IL-4 is known to signal via two distinct receptor complexes, signalling predominantly via the JAK1/STAT6 pathway. The high-affinity Type I IL-4 receptor complex is an IL-4R⍺:γc heterodimer that docks JAK1 and JAK3. In contrast, the low-affinity Type II IL-4 receptor is an IL-4R⍺:IL-13R⍺1 complex, capable of additionally binding IL-13. The Type II complex is believed to further dock JAK2 and engage STAT3 under certain conditions, although the function and context under which this alternative pathway is recruited is unknown^16–20^. Finally, IL-13R⍺2, the high-affinity cognate receptor for IL-13, interacts with the IL-4R⍺ cytoplasmic domain in a mutually antagonistic relationship that ultimately restricts IL-4 signalling^21^.

Studies in our laboratory and elsewhere have shown that differential IL-4/IL-13 receptor representation can have drastic implications on immune outcomes^20,22–25^. Overexpression of IL-4R⍺ and engagement of STAT6 in M2 macrophage have been linked to liver fibrosis and heightened hepatic inflammation^26^. Similarly, overexpression of IL-13R⍺2 in certain cancers have also been reported^27,28^. Moreover, Wijesundera et *al.* have shown down-regulation of IL-4R⍺ expression was associated with enhanced IFN-γ and TNF-⍺ expression by effector CD8 T cells (bettered poly-functionality) following viral infection/vaccination^29^. Recent viral vector-based vaccine studies by Roy et *al.* have also revealed swift regulation of IL-13R⍺2 on lung conventional dendritic cells (cDCs), occurring in both an IL-13 concentration- and STAT3-dependent manner^20^.

Therefore, in this study, we aimed to further unravel the molecular mechanisms underpinning IL-4 regulation in T cells. Specifically, and informed by our recent IL-13/IL-13R⍺2/STAT3 regulatory mechanism identified in cDCs^20^, we endeavored to evaluate whether both STAT3 and STAT6 are also involved in coordinating IL-4 signalling in T cells. These insights may help uncover the molecular basis for specific T and B cell immune outcomes under different viral infections and vaccinations^10,13,14,20,30^, as well as inform rational vaccination design and therapies for diseases underpinned by IL-4/IL-13 dysregulation (e.g. asthma and some cancers^21,27,31–34^).

## Results

### Time- and concentration-dependent IL-4R⍺ regulation observed in both splenic CD4 and CD8 T cells following IL-4 stimulation

**Figure 1:**
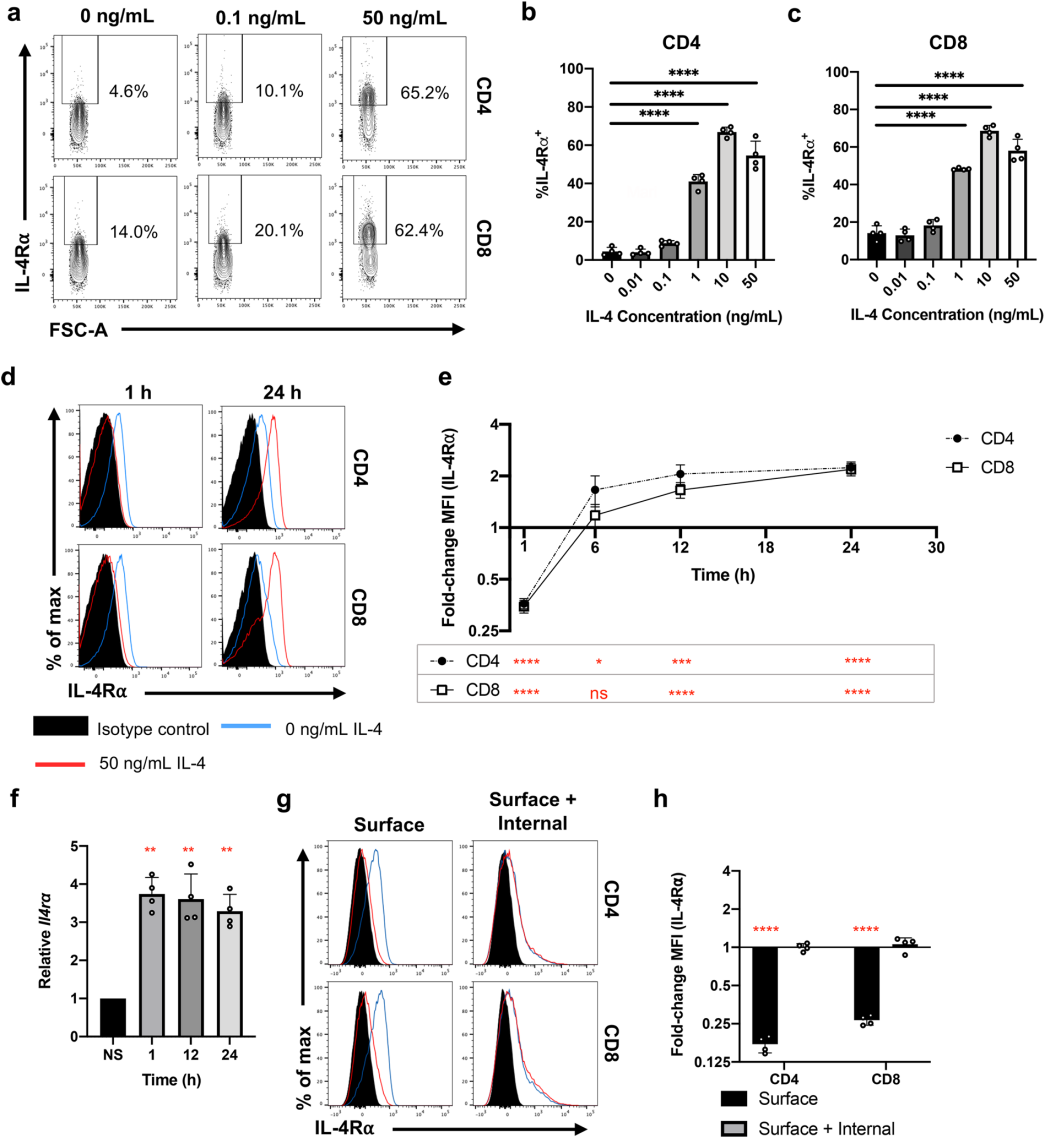
IL-4 stimulation modulates the expression of IL-4R⍺. Whole spleen homogenate derived from naïve 6–8-week-old female BALB/c mice (*n* = 4) were stimulated with recombinant IL-4 in vitro prior to flow cytometric analysis. **(a)** Representative contour plots showing the expression of IL-4R⍺ on pre-gated CD4 and CD8 T cells treated with variable IL-4 concentrations for 24 h. The proportion of both **(b)** CD4 and **(c)** CD8 T cells deemed IL-4R⍺^+^. **(d)** Representative histograms showing the distribution of IL-4R⍺ expression on pre-gated T cells following 50 ng/mL IL-4 stimulation for 1–24 h. **(e)** Fold-change in MFI (IL-4R⍺) with respect to time. **(f)** 100 CD3^+^CD4^+^CD8^-^IL-4R⍺^+^ cells were sorted following 50 ng/mL IL-4 stimulation and qPCR was conducted to measure the transcript levels of *Il4ra*, Ct values are normalised the endogenous *L32* control and represented as 2^-ΔΔCt^ according to the respective non-stimulated (NS) control. **(g)** Representative histograms comparing the surface and total (surface and internal) IL-4R⍺ following 1 h IL-4 stimulation. Notably, total cell IL-4R⍺ was measured by staining after fixation and permeabilisation. **(h)** Fold-change MFI (IL-4R⍺) between cells treated with and not treated with IL-4. Data are represented as mean + SD, with dots representing biological replicates from a single experiment. The experiments were at least repeated at least twice. One-way ANOVA combined with a post-hoc pairwise comparison was conducted (using Prism 8) (black). Paired two-way t-tests were conducted to establish statistical significance in fold-change MFI (red) under each condition. *p*-value denotations: ‘ns’ *p* ≥ 0.05, ‘*’ *p* < 0.05, ‘**’ *p* < 0.01, ‘***’ *p* < 0.001 and ‘****’ *p* < 0.0001.

In this study, whole splenocytes were stimulated for 24 h with varying concentrations of IL-4 to mimic the cytokine environment during infection/vaccination^14,25,35^. IL-4R⍺ expression profiles on both CD4 and CD8 T cells were evaluated by flow cytometry (Fig. 1a). In both T cell subsets, the proportion of cells expressing IL-4R⍺ significantly increased with 1, 10 and 50 ng/mL IL-4, unlike the lower concentrations (Fig. 1b,c). These trends were also reflected in the fold-change mean fluorescent intensity values (MFI), normalised to the respective non-stimulated control (Sup. Fig. 1a–c). Interestingly and consistent with previous findings, IL-4-induced IL-4R⍺ up-regulation was not universal across immune cell types^25^, as typified by splenic MCH-II^+^CD11c^+^ antigen presenting cells (Sup. Fig. 1d,e). Furthermore, IL-13 stimulation did not impact T cell IL-4R⍺ expression, despite the shared receptor system (Sup. Fig. 1f–h).

Data also revealed that the IL-4R⍺ expression was differentially regulated over time following IL-4 stimulation (Fig. 1d,e), as the splenic CD4 and CD8 T cells dramatically down-regulated receptor expression at 1 h, which was subsequently up-regulated at ~ 12 h, and maintained for the remainder of the timecourse. Similarly, significant up-regulation of the *Il4ra* transcript was observed in sorted CD3^+^CD4^+^CD8^-^IL-4R⍺^+^ splenic T cells post IL-4 stimulation, including at 1 h (*p* < 0.01 for all) (Fig. 1f). In contrast, expression of γc and IL-13R⍺1 was mainly down-regulated upon IL-4 encounter (Sup. Fig. 2). Moreover, intracellular and extracellular IL-4R⍺ expression profiles revealed that down-regulation of IL-4R⍺ at 1 h was associated with receptor internalisation (Fig. 1g,h), as no change in the total IL-4R⍺ expression was observed on both T cell subsets (*p* = 0.9815 [CD4]; *p* = 0.4295 [CD8]). This may explain the observed downward trend in γc and IL-13R⍺1 expression following cytokine stimulation; that is, whole receptor complexes were being internalised.

**Figure 2:**
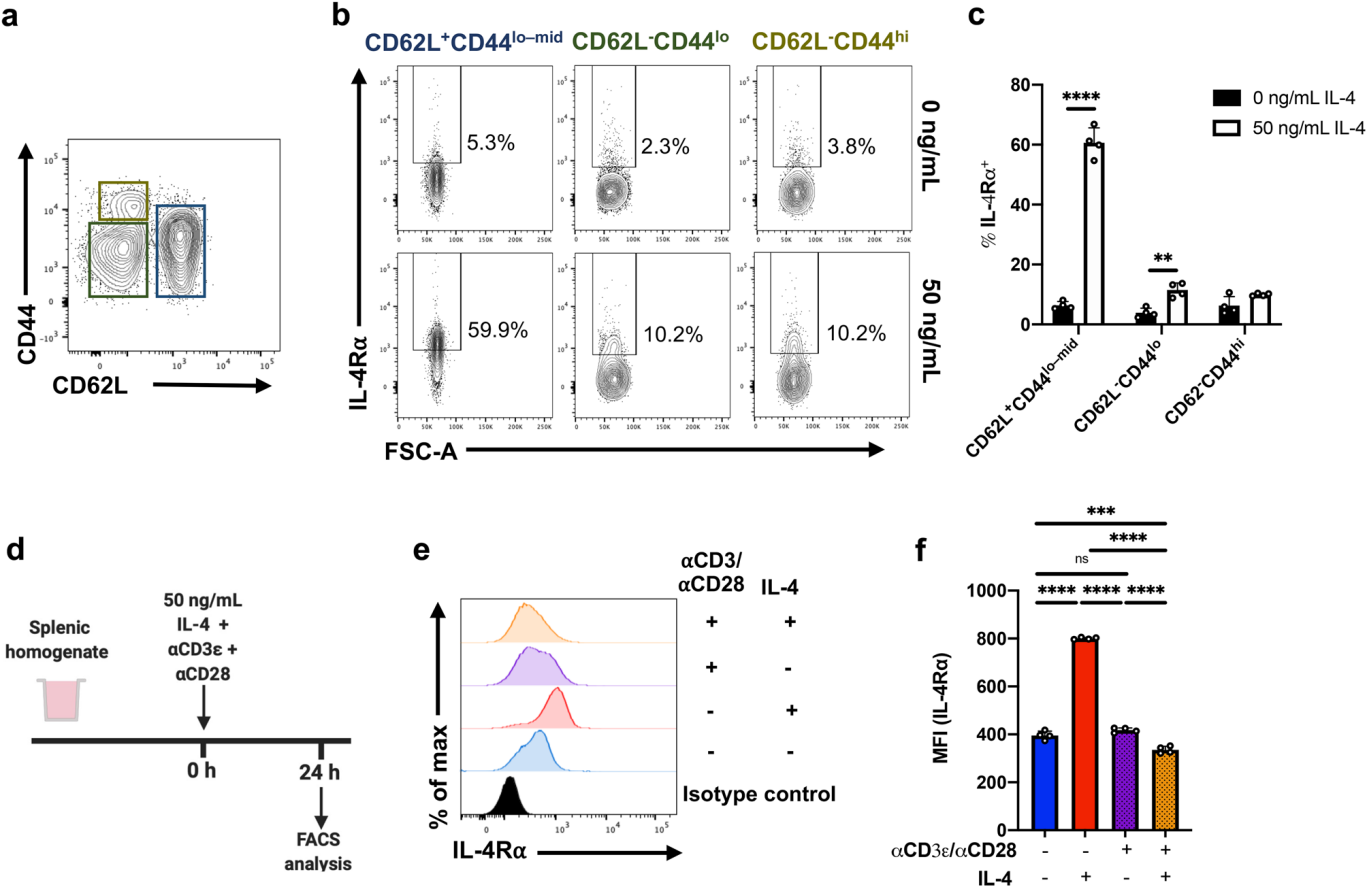
IL-4R⍺ regulation is dependent on CD4 T cell CD62L:CD44 status. **(a)** Whole spleen homogenate derived from naïve 6–8-week-old female BALB/c mice (*n* = 4) were stimulated with 50 ng/mL IL-4 in vitro for 24 h prior to flow cytometric analysis. CD4 T cells were partitioned based on their CD62L:CD44 statuses. **(b)** Representative contour plots show the expression of IL-4R⍺ among CD4 T cells, with respect to CD62L and CD44. **(c)** The percentage of cells deemed IL-4R⍺^+^ following IL-4. **(d)** Splenic homogenate was seeded with ⍺CD3ε and ⍺CD28 and stimulated with 50 ng/mL of IL-4 for 24 h. **(e)** Representative histograms showing the expression profile of IL-4R⍺ on CD4 T cells and **(f)** MFI (IL-4R⍺) for each treatment condition was evaluated. Experiments were repeated three times. Two-way ANOVA combined with Tukey’s post-hoc multiple comparison test was conducted to compare groups. *p*-value denotations: ‘ns’ *p* ≥ 0.05, ‘*’*p* < 0.05, ‘**’*p* < 0.01, ‘***’*p* < 0.001 and ‘****’*p* < 0.0001.

Knowing there can be site-specific IL-4 responses^11,13,36,37^, we also compared IL-4R⍺ profiles between splenic and lung T cells (Sup. Fig. 3). Despite no difference in IL-4R⍺ expression post IL-4 encounter between these two compartments (Sup. Fig. 3a–c), expression of IL-13R⍺2 (the IL-4R⍺ antagonist^21^) was vastly different (Sup. Fig. 3d). Specifically, the baseline proportion of T cells that expressed IL-13R⍺2 was ~ 2.5-fold greater in lung-derived total T cells compared to the spleen (*p* < 0.0001). However, significant down-regulation of IL-13R⍺2 was observed on lung T cells following IL-4 stimulation compared to unstimulated cells (*p* < 0.0001), a change not observed in splenic T cells (*p* = 0.3473).

**Figure 3:**
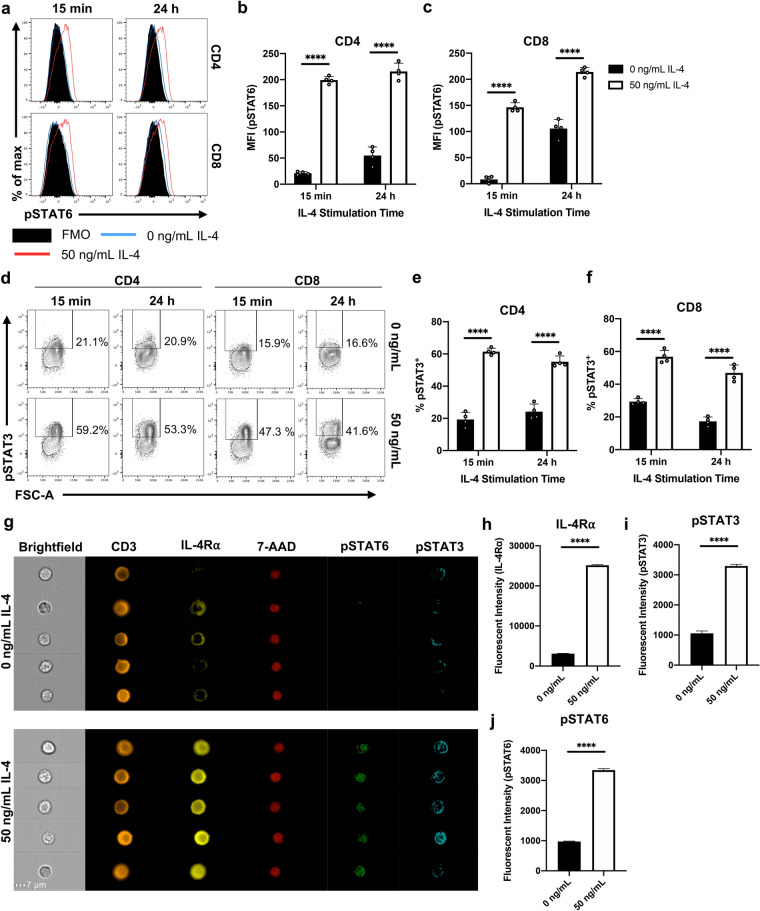
IL-4 stimulation promotes phosphorylation of both STAT6 and STAT3 on T cells. Whole spleen homogenate derived from naïve 6–8-week-old female BALB/c mice (*n* = 4) were stimulated with 50 ng/mL IL-4 in vitro for 15 min or 24 h prior to flow cytometric analysis. **(a)** Representative histograms show the expression of pSTAT6. MFI (pSTAT6) of both **(b)** CD4 and **(c)** CD8 T cells between treatment conditions. **(d)** Representative contour plots showing the expression of pSTAT3; since pSTAT3 expression was bimodal, MFI was not evaluated. The proportion of **(e)** CD4 and **(g)** CD8 T cells that were deemed pSTAT3^+^. Data are represented as mean + SD, with dots representing biological replicates from a single experiment. The experiments were repeated three times. Two-way ANOVA combined with Tukey’s post-hoc multiple comparison test was conducted to compare groups. **(g)** Whole spleen homogenate derived from naïve 6–8-week-old female BALB/c mice were stimulated with 50 ng/mL IL-4 in vitro for 24 h prior to ImageStream analysis. Representative images of pre-gated total T cells. Note that cells were fixed, allowing 7-AAD to be used as a nuclear stain. The fluorescent intensity of **(h)** IL-4Ra, **(i)** pSTAT6 and **(j)** pSTAT3 were compared between treatment groups. Data are represented as mean + SEM, where *n* > 1200 from a single experiment. The experiment was repeated three times. Student’s t-tests were conducted to compare groups. *p*-value denotation: ‘****’*p* < 0.0001.

### IL-4R⍺ regulation mainly occurs on naïve T cells

To further dissect IL-4R⍺ regulation, CD4 T cells were also assessed according to their effector status (CD62L and CD44 expression) (Fig. 2a). Data revealed that IL-4 stimulation promoted the CD62L^+^CD44^lo–mid^ (antigen naive) CD4 T cell population to upregulate the proportion of IL-4R⍺^+^ cells by ~ eightfold compared to the unstimulated control (*p* < 0.0001) (Fig. 2b,c). In contrast, the CD62L^-^CD44^lo^ (effector) population showed a gain of ~ twofold (*p* = 0.008), whilst the CD62L^-^CD44^hi^ (memory) population was unaffected (*p* = 0.202).

To further understand the implications of T cell activation in IL-4R⍺ regulation on T cells, splenocytes were exposed to ⍺CD3ε and ⍺CD28 (Fig. 2d). Interestingly, while ⍺CD3ε and ⍺CD28 failed to elicit changes in IL-4R⍺ expression compared to non-stimulated cells, in combination with IL-4, the receptor was modestly down-regulated (*p* < 0.0001) (Fig. 2e,f). Interestingly, when the above experiment was performed using sorted naïve CD4 T cells, IL-4R⍺ was found to be upregulated independent of concurrent TCR stimulation (Sup. Fig. 4). This indicated that secondary cytokines (e.g. TNF-⍺ and/or IFN-γ) expressed by other functional cell types, such as effector/memory T cells, could also be associated with IL-4R⍺ regulation on naïve T cells.

**Figure 4:**
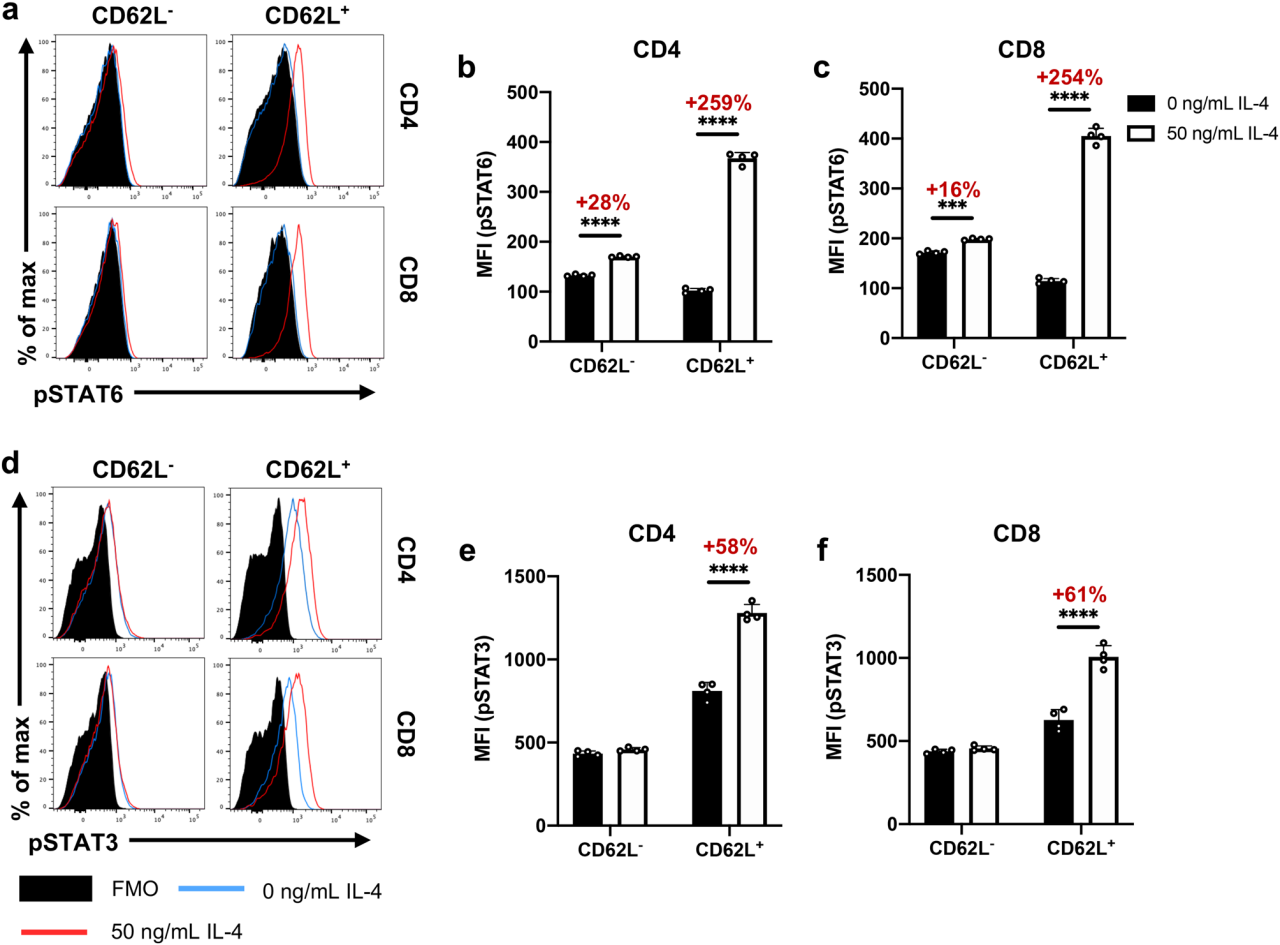
IL-4-induced activation of STAT6 and STAT3 contingent on T cell CD62L status. **(a)** Whole spleen homogenate derived from naïve 6–8-week-old female BALB/c mice were stimulated with 50 ng/mL IL-4 in vitro for 24 h prior to flow cytometric analysis. Representative histograms showing pSTAT6 expression among pre-gated CD4 and CD8 T cells partitioned based on CD62L status. MFI (pSTAT6) of both **(b)** CD4 and **(c)** CD8 T cells. Similarly, the expression of pSTAT3 among T cell subsets with respect to CD62L was evaluated **(d–f)**. Data are represented as mean + SD, where dots represent biological replicates from a single experiment. The experiments were repeated at least twice. Two-way ANOVA combined with Tukey’s post-hoc multiple comparison test was conducted to compare groups. *p*-value denotation: ‘***’*p* < 0.001 and ‘****’*p* < 0.0001.

### IL-4 promotes not only STAT6 but also STAT3 activation in naïve T cells

Our recent studies have shown that lungs DCs respond to different IL-13 conditions by modulating IL-13 receptors, STAT3/STAT6 phosphorylation and TGF-β1 expression^20^. Knowing IL-4 and IL-13 share a common receptor system, we next evaluated the phosphorylation status of both STAT6 and STAT3 on splenic CD4 and CD8 T cells following 15 min (previously shown to cause an optimum fold-change in pSTAT6)^38^ and 24 h IL-4 stimulation (Fig. 3a). At both time points, highly significant increases in STAT6 phosphorylation (pSTAT6) (Fig. 3b,c), as well as pSTAT3, was detected in both T cell subsets (p < 0.0001 for all) (Fig. 3d–f). ImageStream analysis was performed to assess the subcellular localisation of IL-4R⍺, pSTAT3 and pSTAT6 in splenic T cells, comparing IL-4 stimulation conditions (Fig. 3g). The stimulated group significantly increased the expression of these makers, as shown by the elevated fluorescence intensity values between the two groups (*p* < 0.0001; *n* < 1200 cells) (Fig. 3h–j). Interestingly, IL-4 stimulated cells showed a higher degree of pSTAT6 nuclear localisation when compared to the 7-AAD nuclear dye control (Fig. 3g). However, surprisingly, pSTAT3 appeared to be less densely trafficked to the nucleus compared to pSTAT6 (Fig. 3g). The nuclear localisation of pSTAT3 may be a time-dependent and may be linked to an active regulation of the two STAT molecules according to the IL-4 environment, and thus warrant further investigation.

Our findings also revealed that the IL-4-induced phosphorylation of STAT3 and STAT6 were also linked to the T cell effector status (CD62L expression) (Fig. 4), similar to what was observed with IL-4R⍺ regulation (Fig. 2). Specifically, change in pSTAT6 MFI was ~ tenfold greater in CD62L^+^ CD4 and CD8 T cells compared to the CD62L^-^ population (Fig. 4a–c). Similarly, significant enhancement of pSTAT3 MFI was only detected in the CD62L^+^ but not the CD62L^-^ subsets (Fig. 4d–f). It is also noteworthy that the upregulation of pSTAT6 MFI was found to be IL-4 concentration dependent, with phosphorylation detected from 1 to 50 ng/mL (Sup. Fig. 5a,b). However, regulation of pSTAT3 MFI required much greater IL-4 levels, first being detected at 10 ng/mL and maximally observed at 50 ng/mL (Sup. Fig. 5c–d). Thus, for optimal STAT3 phosphorylation, higher IL-4 concentration was used throughout the study. It is noteworthy that t-tests reveal no significant difference in the IL-4Rα MFI in cells with 10 or 50 ng/mL concentrations (CD4: *p* = 0.153, CD8: *p* = 0.958) (Sup. Fig. 1b–c). Interestingly, T cells stimulated with IL-13 (10 ng/mL) did not show any STAT6 or STAT3 phosphorylation (Sup. Fig. 5). Moreover, when splenic T cells were stimulated with 50 ng/mL IL-4, CD62L^+^CD44^lo–mid^ CD4 T cells down-regulated TGF-β1 expression, compared to the unstimulated control (*p* = 0.0018) (Sup. Fig. 6). Interestingly, the CD62L^-^ CD4 T cell subsets did not down-regulate TGF-β1, which is consistent with the STAT3 and STAT6 phosphorylation profiles observed with CD62L^+^ and CD62L^-^ T cell subsets (Sup. Fig. 6; Sup. Fig. 5).

**Figure 5:**
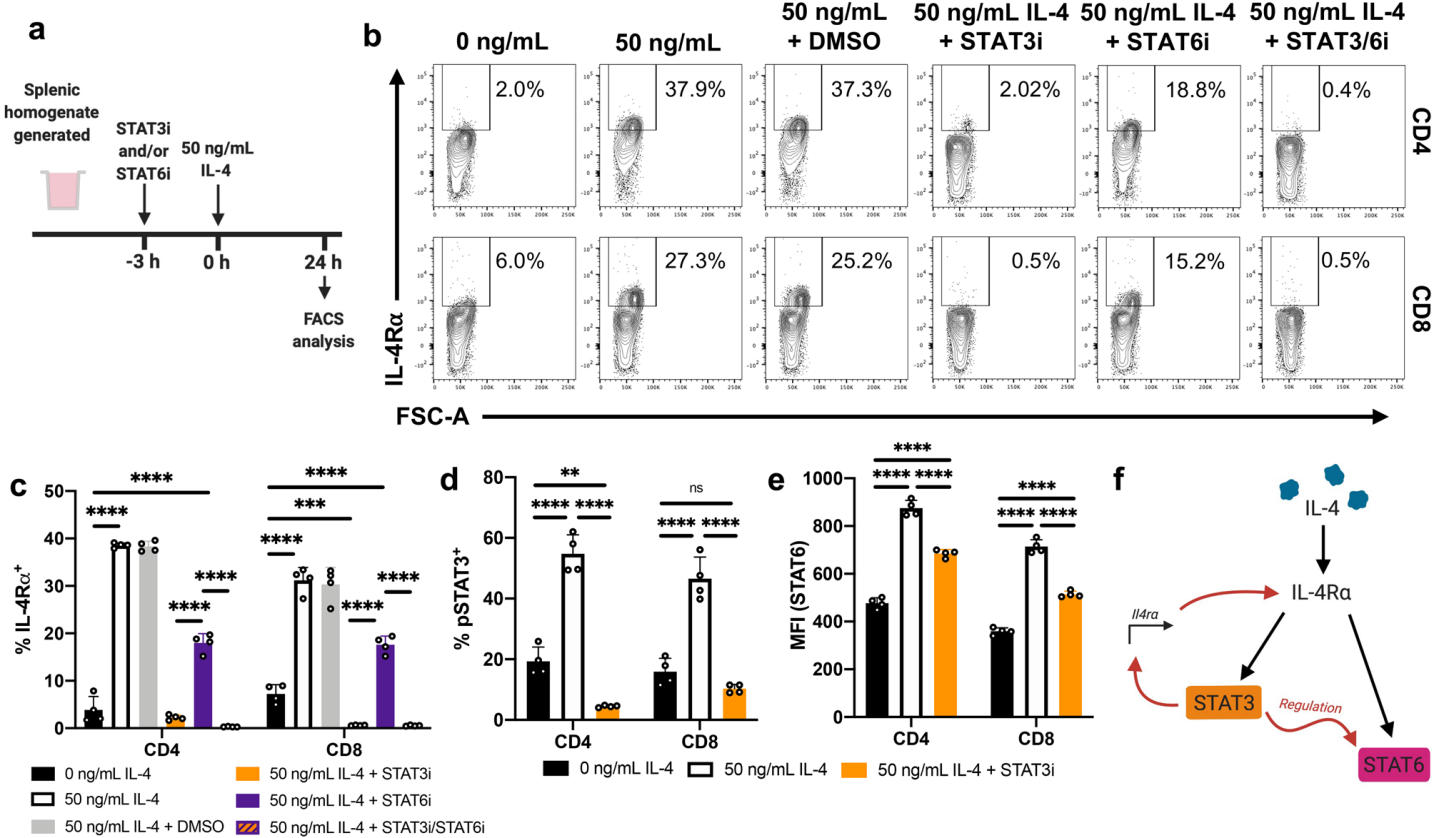
STAT3 regulates IL-4R⍺ expression to affect downstream STAT6 activation. **(a)** Whole spleen homogenate derived from naïve 6–8-week-old female BALB/c mice (*n* = 4) were pre-incubated with STAT3i and/or STAT6i in vitro for 3 h prior to stimulation with 50 ng/mL IL-4 for 24 h prior to flow cytometric analysis. **(b)** The percentage of CD4 and CD8 T cells found to be IL-4R⍺^+^ for each inhibitory/stimulatory group. **(c)** Representative contour plots showing the expression of IL-4R⍺. **(d)** Cells were pre-incubated with STAT3i prior to 24 h IL-4 stimulation. Phosphorylation statuses were subsequently evaluated. The percentage of T cells found to be pSTAT3^+^. **(e)** pSTAT6 MFI for each treatment group. Representative plots shown in Sup Fig. 5e–f. **(f)** Graphical and temporal depiction of the proposed regulatory relationship between STAT3 and STAT6––created using BioRender. Data are represented as mean + SD, where dots represent biological replicates from a single experiment. The experiments were repeated at least twice. Two-way ANOVA combined with Tukey’s post-hoc multiple comparison test was conducted to compare groups. *p*-value denotations: ‘ns’ *p* ≥ 0.05, ‘**’*p* < 0.01, ‘***’*p* < 0.001 and ‘****’*p* < 0.0001.

**Figure 6:**
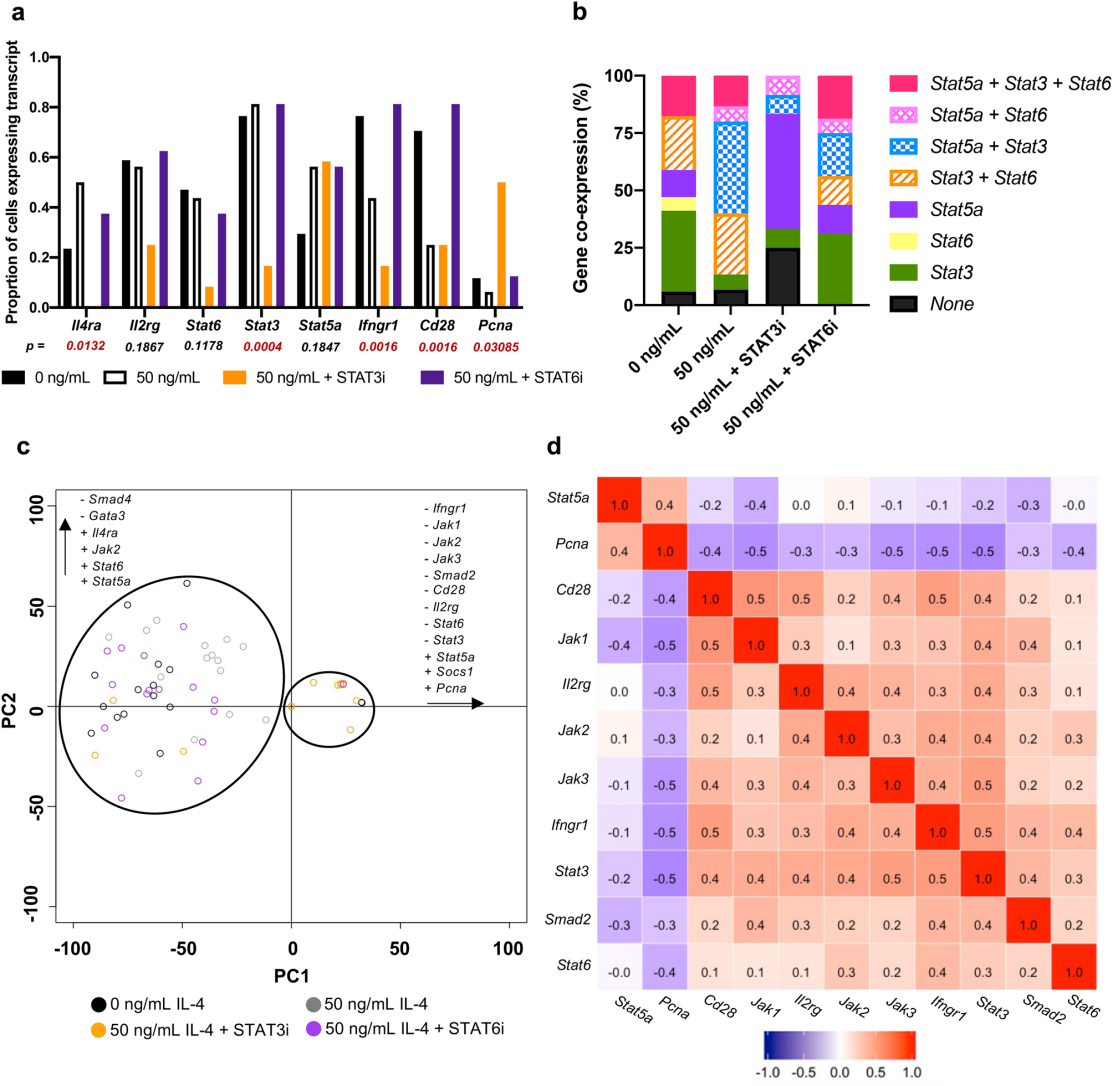
Expression of IL-4/IL-13-associated genes requires STAT3 availability. Whole spleen homogenate derived from naïve 6–8-week-old female BALB/c mice were pre-incubated with STAT3i or STAT6i in vitro for 3 h prior to stimulation with 50 ng/mL IL-4 for 24 h. Fluidigm Biomark 48.48 analyses were performed on single CD3^+^CD4^+^CD8^-^IL-4R⍺^+^ cells, evaluating transcript expression of genes-of-interest. **(a)** dichotimised gene expression profiles of genes-of-interest between treatments. Statistical significance was determined by performing a Fisher’s exact test. (**b**) Co-expression analysis of members of the STAT family (STAT6, STAT3 and STAT5a). **(c)** PCA was conducted to evaluate heterogeneity in transcriptional profiles, particularly whether the heterogeneity can be explained by the treatment (refer Sup. Fig. 7a,b for loadings). **(d)** For genes expressed by at least 15% of cells, Spearman’s rank correlation analysis was performed to evaluate correspondence of genes pairwise. According to the similarity in transcriptional profiles, a major cluster of positively correlated genes were identified and highlighted, along with a negatively correlated cluster (refer Sup. Fig. 7a for dendrogram). The experiment was repeated twice, pooling the data. *n* ~ 15 cells per group.

### STAT3 inhibition down-regulates IL-4R⍺ and STAT6 phosphorylation

Knowing STAT6 or STAT3 were phosphorylated following IL-4 stimulation, their impact on IL-4R⍺ regulation was evaluated using small-molecule inhibitors as described previously (Fig. 5a)^20^. STAT6 inhibition partially down-regulated (halved) the IL-4R⍺ expression on IL-4-stimulated CD4 and CD8 T cells, compared to the uninhibited control (*p* < 0.0001) (Fig. 5b,c). However, and remarkably, drastic down-regulation of IL-4R⍺ was observed on both subsets following STAT3 inhibition (*p* < 0.0001); combined STAT6/STAT3 inhibition showed a similar response to STAT3 only inhibition (Fig. 5b,c). Next, phosphorylation analyses following IL-4 stimulation and STAT3 inhibition revealed differential IL-4-inducible STAT3 and STAT6 phosphorylation (*p* = 0.0001) (Fig. 5d,e; Sup. Fig. 6e,f). Specifically, STAT3 inhibition limited STAT6 phosphorylation by ~ 50% in both CD4 and CD8 T cell subsets (Fig. 5f).

### STAT3 inhibition also modulates other biomarkers associated with IL-4/IL-13 signalling on CD4^+^IL-4R⍺^+^ T cells

Fluidigm 48.48 Biomark analysis was performed on single CD3^+^CD4^+^CD8^-^IL-4R⍺^+^ T cells to compare the transcriptional profiles of IL-4/IL-13 signalling-associated markers following STAT3/STAT6 inhibition and IL-4 stimulation, as described in the methods. Transcriptional expression of 42 genes were evaluated (Sup. table 1). Subsequent analyses were only performed on 16 genes that met the inclusion criterion: at least 15% of all cells expressing a given transcript (Sup. Fig. 7a). Dichotomised transcript expression analysis of the select genes showed unique expression profiles according to the treatment (Fig. 6a; Sup. Fig. 7a–c). For example, the proportion of cells expressing *Il4ra* increased upon IL-4 stimulation, which was down-regulated following STAT6 inhibition and was completely ablated upon STAT3 inhibition (*p* = 0.0132, Fisher’s exact test) (Fig. 6a). These findings clearly reflected the trends observed at the protein level (Fig. 5c). STAT3 inhibition also downregulated the expression of *Stat3* and *Stat6*, although *Stat5a* remained unaffected (Fig. 6a,b). IL-4 stimulation was associated with reduced expression of *Ifngr1* and *Cd28*, which were recovered following STAT6 inhibition. Interestingly, *Pcna* was predominantly detected on STAT3 inhibited cells, which was expected since STAT3 inhibits T cell proliferation^39^. 100 sorted CD3^+^CD4^+^CD8^-^IL-4R⍺^+^ T cell RT-qPCR further validated the trends of select markers (Sup. Fig. 7d–k).

**Figure 7:**
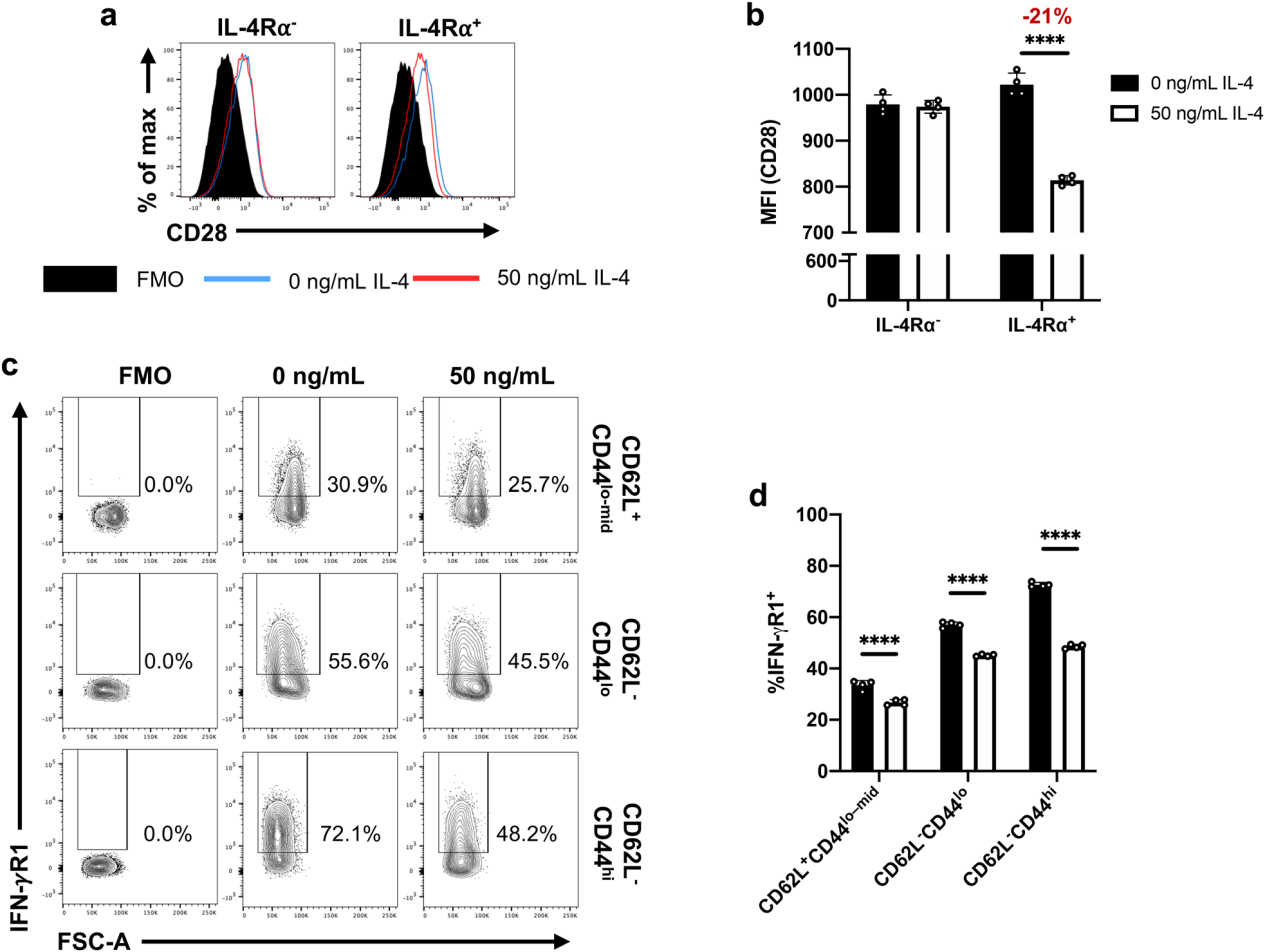
IL-4 stimulation downregulates CD28 and IFN-γR1 expression on CD4 T cells. Whole spleen homogenate derived from naïve 6–8-week-old female BALB/c mice (*n* = 4) were stimulated with 50 ng/mL IL-4 for 24 h. **(a)** Representative histograms showing CD28 expression on pre-gated CD4 T cells partitioned based on their IL-4R⍺ status. **(b)** MFI (CD28) for each treatment condition. **(c)** Representative contour plots showing the expression of IFN-γR1 on pre-gated CD4 T cells for each CD62L:CD44 status. **(d)** The proportion of cells deemed IFN-γR1^+^. Data are represented as mean + SD, where dots represent biological replicates from a single experiment. The experiments were repeated at least twice. Two-way ANOVA combined with Tukey’s post-hoc multiple comparison test was conducted to compare groups. *p*-value denotation: ‘****’ *p* < 0.0001.

Principle component analysis (PCA) was conducted on the 16 select genes, where expression was normalised to the *L32* housekeeping gene control, as described previously^40^. The first four PCs accounted for over 83% of the variance, suggesting strong correlations between biomarkers. PC1 accounted for the majority of the variance (54%), with treatment playing a significant role (ANOVA: *p* = 0.00066). Evaluation of PC1 scores revealed that the STAT3i-treated cells clustered away from the other treatment groups (non-stimulated: *p* = 0.0016, IL-4 stimulated: *p* = 0.0199; and IL-4 stimulated, STAT6-inhibited: *p* = 0.0011) (Fig. 6c). When considering the loadings of these PCs (Sup. Fig. 8a–b), it further corroborated with the trends inferred by the dichotomised data. The transcriptional profile of the STAT6-inhibited cells was less severely affected compared to the STAT3-inhibited cells; this may be a consequence of the cells being IL-4R⍺^+^, which are likely naïve T cells (Fig. 2) and thus do not have access to GATA3-mediated transcriptional activity. Spearman’s rank correlation test was performed to evaluate similarity in the expression profiles between markers (Fig. 6d; Sup. Fig. 8c). The major cluster of positively correlated genes consisted of *Cd2*8, *Jak1*, *Jak2*, *Ifngr1*, *Stat3*, *Il2rg*, *Smad2* and *Stat6*, as reflected by the rank correlation-derived dendrogram (Sup. Fig. 8d). Notably, the expression of these markers was typically down-regulated upon STAT3 inhibition, according to the dichotomised outputs (Sup. Fig. 7a). Inversely correlated to this cluster were *Stat5a* and *Pcna*, both of which were highly expressed upon STAT3 inhibition.

**Figure 8:**
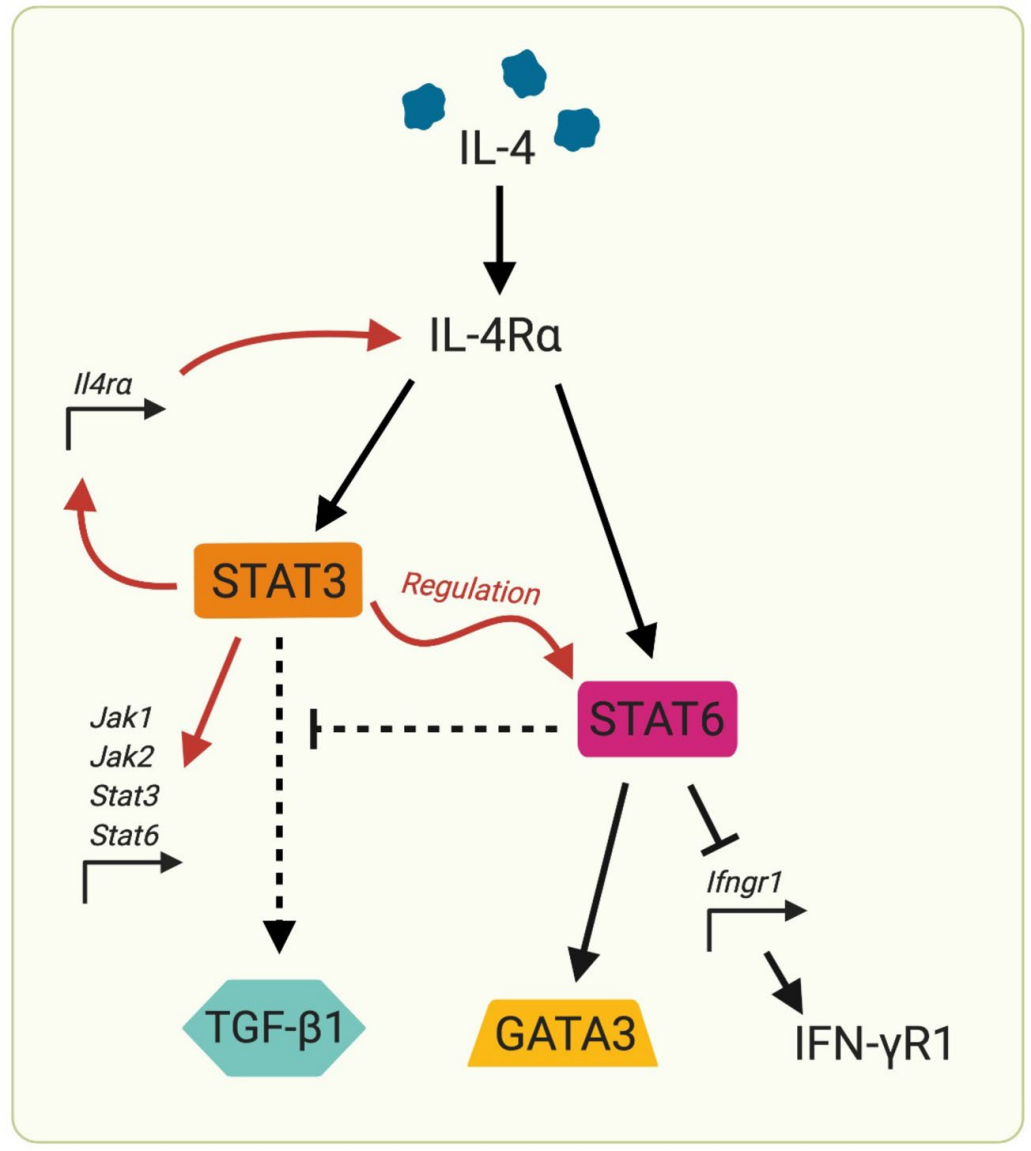
STAT3 co-ordinates downstream IL-4 signalling outcomes in T cells. Studies have shown, for the first time, that STAT3 signalling capability is critical to regulating T cell supply of IL-4R⍺ and, by extension, responsiveness to the IL-4 environment. Subsequently, inhibition of STAT3 resulted in ablated STAT6 engagement. We suspect this affects IL-4 signalling outcomes, including the production of TGF-β1, GATA3 and IFN-γR1. Collectively, this molecular mechanism may have profound implications for T cell fate and function during infection/vaccination. Created using BioRender.

### IL-4 stimulation affects CD28 and IFN-γR1 expression on T cells

Fluidigm analysis revealed that IL-4 stimulation down-regulated the expression of *Cd28* and *Ifngr1* on CD3^+^CD4^+^CD8^-^IL-4R⍺^+^ cells (Sup. Fig. 7a). When the respective protein products were evaluated on splenic CD4 T cells 24 h post IL-4 stimulation, down-regulation of the co-stimulatory receptor, CD28, in IL-4R⍺^+^ (*p* < 0.0001) was observed, although not in the IL-4R⍺^-^ subset (*p* = 0.914) (Fig. 7a,b). In contrast, the down-regulation of IFN-γR1 expression was independent of CD62L/CD44 status (*p* < 0.0001) (Fig. 7c,d).

## Discussion

Mechanisms by which IL-4 is regulated in naïve, effector and memory T cell subsets still remain elusive. In the current study, we identify that antigen-naïve (CD62L^+^CD44^lo–mid^) CD4 and CD8 T cells can significantly up-regulate IL-4R⍺ expression in an IL-4 concentration- and time-dependent manner. Moreover, STAT6 inhibition partially reduced the proportion of CD4 and CD8 T cells expressing IL-4R⍺, whilst STAT3 and/or combined STAT3/STAT6 inhibition completely abolished IL-4-inducible IL-4R⍺ activity. STAT3 inhibition also prevented IL-4-induced STAT6 phosphorylation, suggesting a novel receptor-mediated inter-regulatory relationship between the two transcription factors. Furthermore, inhibition of STAT3 down-regulated *Ilr4a*, *Il2rg*, *Jak1*, *Jak2*, *Stat3* and *Stat6* gene expression in IL-4R⍺^+^ CD4 T cells, indicating that STAT3 broadly regulated genes associated with IL-4/IL-13 signalling. Intriguingly, the engagement of STAT3 was found only under high IL-4 concentrations (at least 10 ng/mL), unlike STAT6 (1 ng/mL). This was consistent with both (1) the existing IL-4 receptor model consolidated by Tabata et *al.*, where the Type II IL-4 receptor complex (IL-4R⍺:IL-13R⍺1) has a lower binding affinity to IL-4 compared to Type I complex (IL-4R⍺:γc)^41,42^, and (2) recent findings by Roy et *al.* revealing IL-13/IL-13R/STAT3 regulatory mechanisms^20^. Notably, previous studies have also described IL-4-associated STAT3 phosphorylation in lymphocytes^43^, despite IL-4 signalling conventionally being linked to the JAK1/STAT6 pathway^4,42^. Jointly, these findings indicated that STAT3 could be the master regulator of IL-4 signalling for naïve T cells under high IL-4 conditions (Fig. 8). This is similar to that observed in lung DCs, where STAT3 maintains IL-13/IL-13R⍺2 homeostasis^20^.

Interestingly, responsiveness to IL-4 was mainly observed in naïve CD4 and CD8 T cells (CD62L^+^CD44^lo–mid^), compared to CD62L^-^ (effector and memory) T cell subsets. This may seem intuitive, given the role of IL-4 in determining naïve T cell fate^44,45^. Despite this study exploring the effects of IL-4 in vitro, the observations correspond with vaccination studies performed by Wijesundara et *al.*, where IL-4R⍺ was down-regulated on viral-specific T cells upon infection/vaccination^25^. Here, we further demonstrated that the CD62L^-^ population was unable to phosphorylate STAT3 upon low or high IL-4 stimulation and, consequently, failed to upregulate IL-4R⍺, compared to naïve T cells. Importantly, the IL-4 environment can also significantly impact the quality/avidity of T cell immunity, where IL-4R⍺ expression was inversely related to IFN-γ and TNF expression on effector T cells following infection/vaccination^11,13,25^. Since we have shown that IL-4 signalling regulation occurs predominantly on naïve T cells, it may indicate that the early IL-4 environment during infection/vaccination may have significant implications in determining T cell fate.

In addition, naïve CD4 T cells also down-regulated the expression of both IFN-γR1 and TGF-β1 upon IL-4 stimulation. These findings may reflect how the cytokine environment upon pathogen encounter (specifically rapid IL-4 secretion by innate immune cells, including eosinophils, basophils, ILCs and macrophages^14,46–50^) modulates downstream immune outcomes. Our findings may suggest that early IL-4 conditions naïve T cell fate. This may explain why elevated serum IL-4 can be detected following certain parasitic, helminthic and viral infections, including SARS-CoV-2^14,46–51^.

Moreover, this study revealed that splenic and lung-derived T cells regulated IL-4R⍺ in a similar manner, even though the expression of IL-13R⍺2 was vastly different between the two compartments. Briefly, the lung T cells showed significantly greater baseline IL-13R⍺2 expression (similar to lung DC) that was uniquely down-regulated upon IL-4 exposure. Interestingly, IL-13 did not impact IL-4/IL-13 receptor regulation in T cells. Knowing that IL-13R⍺2 can inhibit IL-4 signalling by interacting with the cytoplasmic domain of IL-4R⍺ to prevent JAK1 activity^33^, we propose that site-specific expression of IL-13R⍺2 may facilitate tissue-specific IL-4 signalling outcomes. This may explain how, despite sharing a common receptor system, IL-4 and IL-13 have both distinct and overlapping functions in different compartments (mucosal versus systemic). Specifically, this study supports the emerging dichotomy that IL-4 predominantly regulates adaptive cells, whilst IL-13 regulates innate cells at the first line of defence^20^.

Interestingly, our previous studies have demonstrated that 24 h post viral vector-based vaccination, ILC2-derived IL-4/IL-13 uniquely regulated DC activity at the vaccination site, modulating downstream immune outcomes^20,30,36,37,52,53^. Mucosal vaccination can induce lower ILC2-derived IL-4/IL-13 at the vaccination site compared to systemic delivery, with mucosal delivery characterised by high avidity/poly-functional T cells^11,13^. Knowing that IL-4R-antagonising HIV vaccination improves the avidity, poly-functionality and cytotoxicity of CD4 and CD8 T cells in mice and non-human primates^10,14,15,35^, current findings indicate that adjuvants that transiently modulate the STAT3/STAT6/IL-4R⍺ axis may prove useful in fine-tunning pathogen-specific vaccine outcomes. Thus, these IL-4 regulatory mechanisms, particularly the role of STAT3, warrants further investigation in the context of infection/vaccination.

Notably, several alternative IL-4 signalling pathways have long been reported in some cell types^54–59^, however, under which circumstances these get activated in T cells is not well-defined. In the current study, while the genes encoding many of these alternative pathways were investigated (*Irs1*, *Irs2*, *Stat5a* and *Stat1*), *Stat5a* was the only biomarker that was significantly up-regulated in response to IL-4, independent of STAT3 and STAT6. Knowing that IL-4 can trigger the phosphorylation of STAT5 via γc-docked JAK1^55,60^, our findings may infer that STAT5 may be important for IL-4 signalling in T cells, specifically, given that *Stat5a* expression was maintained under STAT3 inhibition. Moreover, our findings also revealed reduced CD28 expression on IL-4R⍺^+^ cells, suggesting a pro-anergic phenotype. Similarly, down-regulated *Pcna* expression was observed following STAT3 inhibition, indicating that STAT3 may also be associated with T cell proliferation, which is consistent with previous findings^39^.

In conclusion, here we provide new insight into how different T cell subsets regulate IL-4 activity under different IL-4 conditions (Fig. 8). We show that naïve T cells directly respond to IL-4 (but not IL-13) in a STAT3/STAT6 dependent manner. Collectively, our findings clearly demonstrate that, similar to IL-13/IL-13R⍺2 signalling in lung cDCs, STAT3 appears to be the master regulator of IL-4/IL-4R⍺ signalling in naïve T cells, allowing them to differentially regulate IL-4R⍺ and modulate downstream immune outcomes. This may implicate T cell fate and, by extension, pathogen immune evasion. We believe our findings have significant potential to inform the design of improved vaccine adjuvants against chronic viral pathogens, as well as therapies against IL-4/IL-13-associated diseases.

## Methods

### Mice and ethics statement

In this study, 6–8-week-old female naïve Charles River WT BALB/c mice were, purchased from the Australian Phenomics Facility at the Australian National University (ANU). WT BALB/c mice were chosen mainly because all our previous in-vivo infection/vaccination studies related to this study were conducted in this strain of mice^10,11,13,15,20^. All animals were maintained, monitored daily, and experiments were performed in accordance with the Australian National Health and Medical Research Council (NHMRC) guidelines within the Australian Code of Practice for the Care and Use of Animals for Scientific Purposes. Animal ethics were approved by the Australian National University’s Animal Experimentation and Ethics Committee (AEEC), ethics protocol number A2017/15. Animals were maintained and used in accordance with the latest ARRIVE guidelines^61^.

### In vitro cytokine and TCR stimulation

Whole spleen or lung single-cell suspensions was prepared, as previously described^30,52,62^. In stimulation assays, whole spleen suspensions or two-step magnetic-activated cell sorted (MACS) naïve CD4 T cells were used. The MACS sorting was performed according to the manufacturer’s instructions (Miltenyi Biotec), where firstly CD4 T cells were negatively selected and subsequently CD62L^+^ cells were positively selected. 1 × 10^6^ cells were stimulated with IL-4 (0.001–50 ng/mL) or 0.1–10 ng/mL of IL-13 was used to stimulate cells for 15 min and/or 24 h. Cells were incubated at 37 °C under 5% CO_2_. To dissect the role of TCR and co-stimulation in regulating IL-4 responses, single cell suspensions were seeded in plates coated with anti-mouse CD3ε (clone: 145-2C11, Biolegend), incubating 3 μg/mL for 18 h at 4 °C. Cells were subsequently seeded along with suspended anti-mouse CD28 (clone: 37.51, Biolegend) to a final concentration of 0.5 µg/mL.

### STAT6 and STAT3 inhibition assays

In order to evaluate the role of STAT3 and STAT6 in the IL-4/IL-13 signalling in T cells, small-molecule inhibitors were used. Briefly, cell suspensions were pre-incubated with 20 μM Stattic (STAT3i) (Axon Medchem) and/or 100 nM AS161749 (STAT6i) (Axon Medchem) in complete RMPI media for 3 h (37 °C, 5% CO_2_), which correspond to biological concentrations with ~ 85% inhibition^63,64^. Both inhibitor concentrations have previously shown to prevent cytokine-induced phosphorylation, with limited cross-talk among other members of the STAT family, as well as being non-toxic to cells^63,64^. Cells were subsequently stimulated with IL-4 as described above.

### Immunostaining, flow cytometry and ImageStream analysis

Following stimulation, cells were washed to remove the media and blocked with anti-mouse CD16/CD32 (clone: 93, Biolegend) for 20 min at 4 °C. T cell immunostaining was subsequently conducted using allophycocyanin (APC)-conjugated anti-mouse CD3 (clone: 17A2, Biolegend), peridinin-chlorophyll-protein (PerCP)-conjugated anti-mouse CD4 (clone: RM4-5), APC eFluor780-conjugated anti-mouse CD8a (clone: 53–7.7, Invitrogen), Pacific Blue-conjugated anti-mouse CD44 (clone: IM7, Biolegend) and fluorescein isothiocyanate (FITC)-conjugated anti-mouse CD62L (clone: MEL-14, Biolegend), as well as one of several markers-of-interest: phycoerythrin (PE)-conjugated anti-mouse IL-4R⍺ (CD124) (clone: I015F8, Biolegend), PE-conjugated anti-mouse γc (CD132) (clone: TUGm2, Biolegend), PE-conjugated anti-mouse IL-13R⍺1 (CD213a1) (clone: 13MOKA, Biolegend), PE-conjugated anti-mouse IFN-γR1 (CD119) (clone: 2E2, eBioscience), or biotinylated anti-mouse IL-13R⍺2 (clone: BAF539, R&D) followed by PE-conjugated streptavidin (Biolegend). When staining with APC-conjugated anti-mouse CD28 (clone: 37.51, Biolegend), FITC-conjugated anti-mouse CD3 (clone: 17A2, Biolegend) was alternatively used. To evaluate antigen presenting cells, alternative lineage markers were used: APC-conjugated anti-mouse MHC-II (I-A/I-E) (clone: M5/114.15.2, Biolegend) and biotinylated anti-mouse CD11c (clone: N418, Biolegend) followed by Brilliant Violet 421-conjugated streptavidin (Biolegend). Cells were fixed with 1.5% paraformaldehyde (PFA). For the staining of intracellular markers, commercial fixation and permeabilization buffers were employed, according to the manufacturer’s instructions (Biolegend). PE-conjugated anti-mouse LAP (TGF-β1) (clone: TW7-16B4, Biolegend) was subsequently stained.

Due to the stability of phospho-epitopes, an alternative staining strategy was applied to stain for pSTAT3 and pSTAT6^65,66^. Cells were pre-fixed with PFA before permeabilisation with ice cold methanol. Cells were subsequently stained with FITC-conjugated anti-mouse CD3 (clone: 17A2, Biolegend), PerCP-conjugated anti-mouse CD4 (clone: RM4-5), APC eFluor780-conjugated anti-mouse CD8a (clone: 53–7.7, Invitrogen), Brilliant Violet 421 anti-mouse phospho-STAT3 (Tyr705) (clone: 13A3–1, Biolegend) and APC-conjugated anti-mouse phospho-STAT6 (Tyr641) (clone: CHI2S4N, Invitrogen).

5 × 10^5^ events per sample was acquired using a BD LSR II flow cytometer (Beckton Dickson) and data analysed using FlowJo v 10.6.1, according to the gating strategy indicated in (Sup. Fig. 8). ImageStream analysis was conducted using the Amnis ImageStream Mark II (Amnis), resuspending samples in 7-AAD (Biolegend) post-fixing to reveal the nucleus. 10 000 events per sample were collected and analysed using the IDEAS software. Representative single colour controls and the gating strategy indicated in (Sup. Fig. 10). Fluorescent intensities of individual cells were compared between treatments.

### Cell sorting for Fluidigm 48.48 Biomark and qPCR assays

Following cytokine stimulation cells were suspended in LIVE/DEAD Fixable Blue (Thermo Fisher Scientific), according to the manufacturer’s instructions. Following immunostaining, live CD3^+^CD4^+^CD8^-^IL-4R⍺^+^ cells were sorted (BD FACS Aria II) directly into pre-amplification mixture. This mixture contained 2 × reaction buffer, SuperScript III RT/Platinum Taq Mix, 0.2X pooled assays (described in Sup. Table 1), SUPERase•In RNase Inhibitor and DEPC-treated water. Sorted cell mixture was centrifuged at 1454×*g* to break the cells and release the mRNA^40^. cDNA was subsequently synthesised according to the thermo-cycling program: 1 × 50 °C for 15 min, 1 × 95 °C for 2 min and 14–20× (95 °C for 15 s and 60 °C for 4 min) (single or 100 cells).

### Real-time qPCR analysis

To validate the primer probes qPCR was performed on 100 cells using TaqMan qPCR mix, which contained 1 µL of gene expression assay (primer probes in Sup. Table 1), 5 µL of 2 × TaqMan Universal PCR master mix, 1 µL cDNA and 4.5 µL DEPC-treated water. Targets were quantified using the 7900HT thermocycler, according to the following program: 1 × 50 °C for 2 min, 1 × 95 °C for 10 min and 40 x (95 °C for 15 s and 60 °C for 1 min). FAM fluorescence was normalised to ROX (6-carboxy-X-rhodamine). SDS v 2.4. was used to obtain cycle threshold (Ct) values.

### Fluidigm 48.48 Biomark gene expression assay

Fluidigm 48.48 Biomark gene expression assays were performed, as described previously^20,40^. Briefly, the integrated fluidic chip (IFC) (Fluidigm) was primed using the IFC Controller MX, according to the manufacturer’s instructions. cDNA was diluted 1:1 with DEPC-treated water and 20× GE Sample Loading reagent was diluted 1:9 in TaqMan PCR Master Mix. Diluted cDNA and loading reagent were combined 1:1 and loaded onto the IFC chip. Primer assays were diluted 1:1 with 2X GE Assay Loading reagent and loaded into the IFC. The sample and assay were subsequently distributed on the chip via the IFC Controller MX loading program and the gene expression assay was performed and analysed using the GE 48.48 Standard.pcl on the Fluidim Biomark. Ct values were acquired from the Fluidigm Biomark, being normalised to ROX.

### Statistical analysis

Marker-positive cells (calculated as a percentage of the parent population) and MFI values were compared between treatment groups by ANOVA combined with post-hoc, unpaired and parametric multiple comparison tests. Expression of a given marker was represented as either the percentage marker-positive of the parent or as the geometric mean fluorescent intensity (MFI). Fold-change MFI was calculated as described elsewhere^25^ and compared via paired Student’s t test. Paired tests were similarly used to compared fold-change in transcript expression (2^-∆∆Ct^ values; normalised to *L32*, the endogenous control). Dichotomised Fluidigm Biomark outputs were compared between treatments by performing Fisher’s exact test. For genes expressed by at least 15% of cells, *L32*-normalised Ct values were evaluated by performing a Spearman’s rank correlation to establish association between gene expression values. This was subsequently used to perform a PCA, as described elsewhere^20,40^.

## Supplementary Information

Supplementary Information.

## Data Availability

The authors declare that all data supporting this study are available in the paper and supplementary files.
