# Supplementary material for: STAT3 determines IL-4 signalling outcomes in naïve T cells
Source: Sci Rep. 2021 May 18;11:10495. doi: 10.1038/s41598-021-89860-7 (PMC8131749; doi:10.1038/s41598-021-89860-7)
Supplement: Supplementary file 1 — Supplementary Information. [file 41598_2021_89860_MOESM1_ESM.pdf]

SUPPLEMENTARY FILES

STAT3 determines IL-4 signalling outcomes in naïve T cells

Lachlan P. Deimel[1,2], Zheyi Li[1], Sreeja Roy[1,3] and Charani Ranasinghe[1]*

Running title: IL-4Rα and STAT3/STAT6 regulation on T cells

*Correspondence: charani.ranasinghe@anu.edu.au

Telephone: +61 2 6125 4706

[1] Molecular Mucosal Vaccine Immunology Group, Department of Immunology and Infectious Disease, The John Curtin School of Medical Research, The Australian National University, Canberra ACT 2601, Australia.

[2] Sir William Dunn School of Pathology, Medical Sciences Division, University of Oxford, Oxfordshire OX1 3RE, UK.

[3] Department of Immunology and Microbial Disease, Albany Medical College, Albany, New York 12208, USA.

Sup fig. 1: IL-4Rα regulation does not occur in APCs nor is it inducible by IL-13 in T cells.

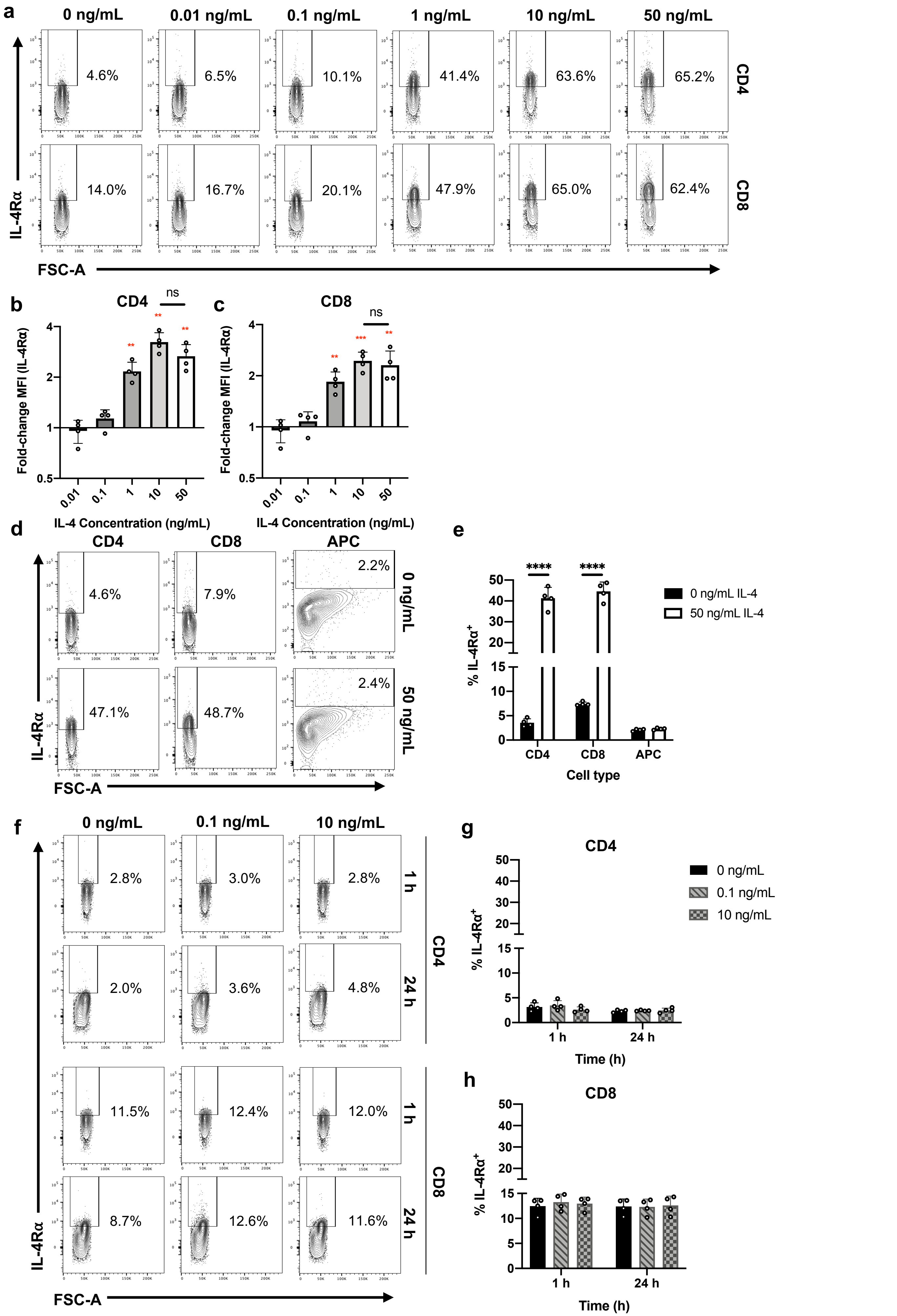

Sup fig. 1: IL-4Rα regulation does not occur in APCs nor is it inducible by IL-13 in T cells. **(a)** Whole spleen homogenate from naïve 6–8-week-old female BALB/c mice were stimulated with variable concentrations of IL-4 (0–50 ng/mL) *in vitro*. The expression of IL-4Rα was evaluated on CD4 and CD8 T cells via flow cytometry. The fold-change in IL-4Rα-associated MFI was compared, normalizing to the respective non-stimulated control **(b–c)**. **(d–e)** The percentage of cells deemed IL-4Rα^{+} following 24 h 50 ng/mL IL-4 stimulation were compared between T cell subsets and APCs. **(f)** Splenic homogenate was alternatively treated with 0.1 or 10 ng/mL of IL-13 *in vitro* for 1 or 24 h and IL-4Rα expression was evaluated. The percentage of IL-4Rα^{+} cells among both **(g)** CD4 and **(h)** CD8 T cells following IL-13 stimulation. Data are represented as mean + SD, where dots represent biological replicates from a single experiment. The experiments were repeated at least twice. Two-way ANOVA combined with Tukey's post-hoc multiple comparison test was conducted to compare groups (black). Paired t-tests were conducted to evaluate the significance in fold-chance (red), *p*-value denotations: 'ns' $p \geq 0.05$, '*' $p < 0.05$, '**' $p < 0.01$, '***' $p < 0.001$ and '****' $p < 0.0001$.

Sup fig. 2: γc and IL-13Rα1 expression is low on CD4 and CD8 T cells and tends to downregulate upon IL-4 stimulation.

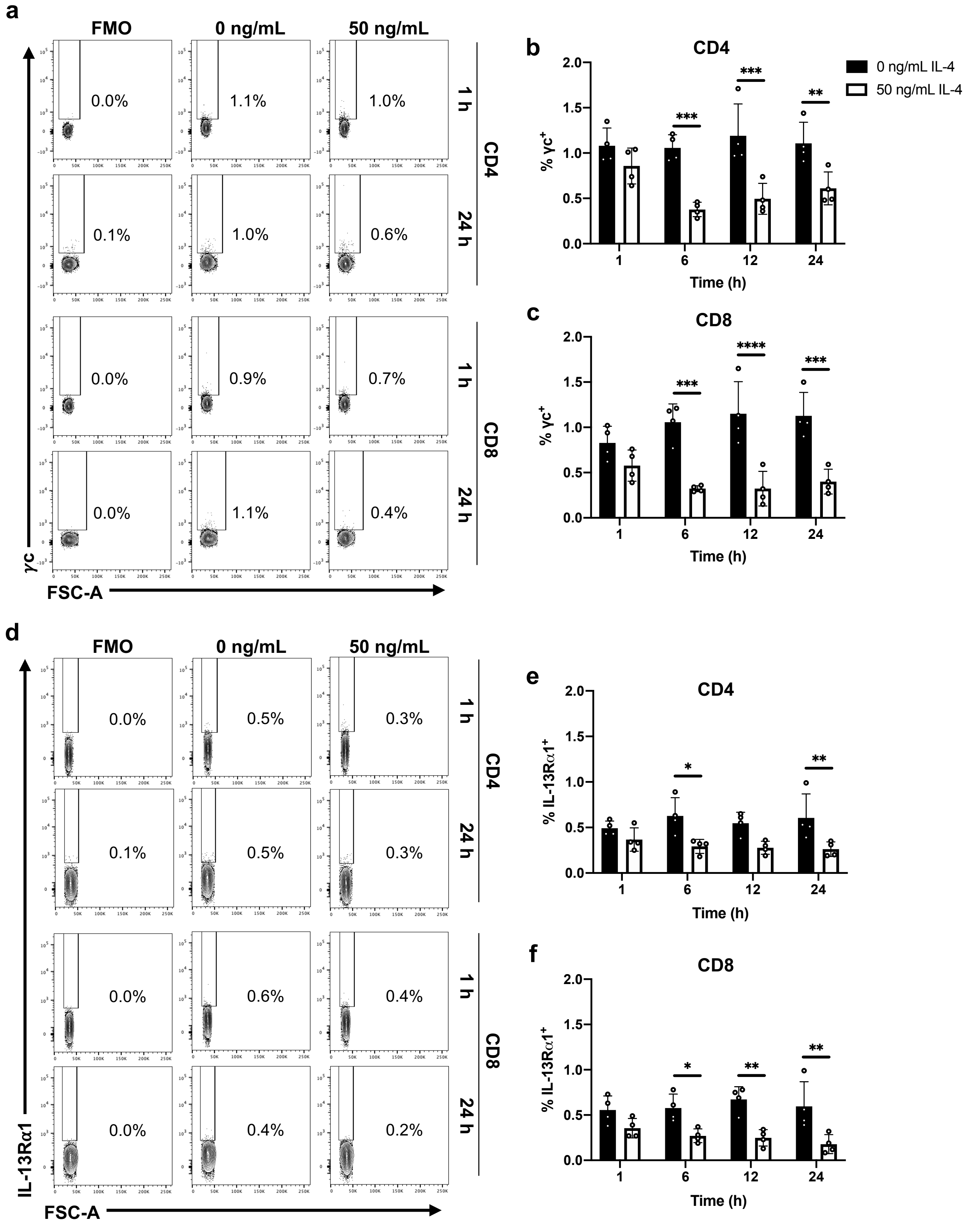

Whole spleen homogenate derived from naïve 6–8-week-old female BALB/c mice were stimulated with 50 ng/mL IL-4 *in vitro* for 1–24 h prior to flow cytometric analysis. **(a)** Representative contour plots showing the expression of γc on pre-gated CD4 and CD8 T cells at both the early (1 h) and late (24 h) timepoints. The percentage of γc^+ **(b)** CD4 and **(c)** CD8 T cells. The expression of IL-13Rα1 was similarly evaluated **(d–f)**. Data are represented as mean + SD, where dots represent biological replicates from a single experiment. The experiments were repeated at least twice. Two-way ANOVA combined with Tukey's post-hoc multiple comparison test was conducted to compare groups. *p*-value denotation: '*' $p < 0.05$, '**' $p < 0.01$, '***' $p < 0.001$ and '****' $p < 0.0001$.

Sup fig. 3: IL-13Rα2 differentially regulated by IL-4 across sites.

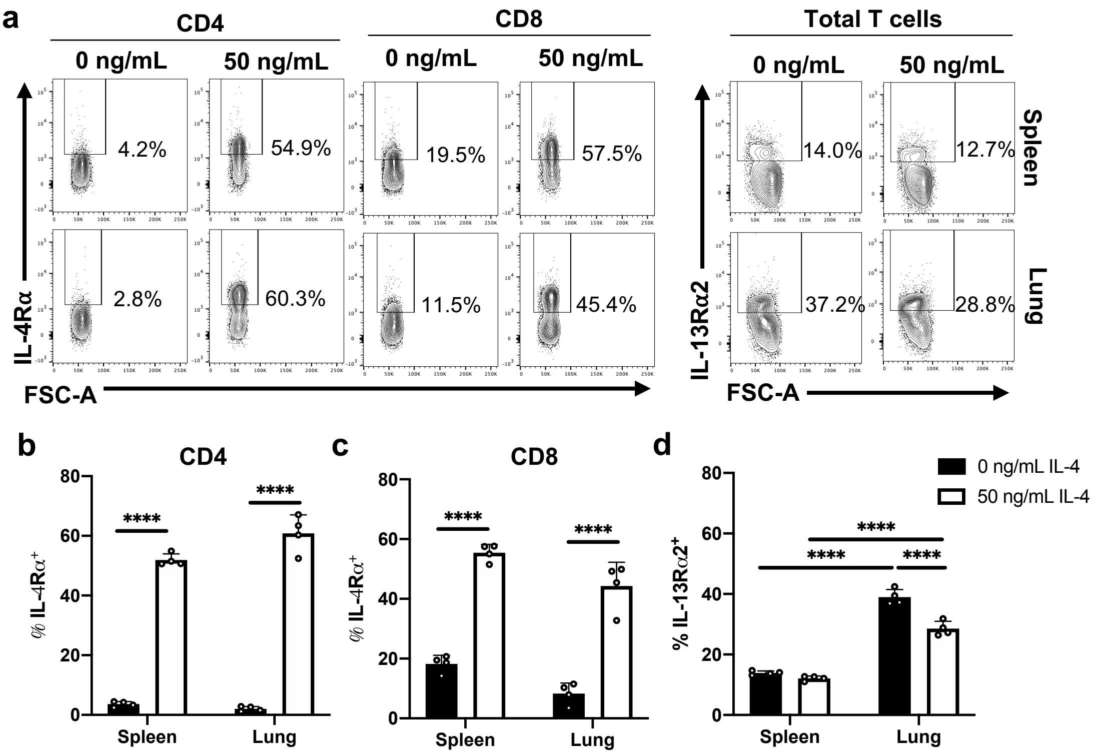

(a) Whole spleen or lung homogenate derived from naïve 6–8-week-old female BALB/c mice were stimulated with 50 ng/mL IL-4 *in vitro* for 24 h prior to flow cytometric analysis. Representative contour plots show the expression of IL-4Rα on pre-gated CD4 and CD8 T cells, as well as IL-13Rα2 on total T cells following IL-4 stimulation. The percentage of cells deemed IL-4Rα$^+$ following IL-4 stimulation on both **(b)** CD4 and **(c)** CD8 T cells. **(d)** Percentage of T cells deemed IL-13Rα2$^+$. Data are represented as mean + SD, where dots represent biological replicates from a single experiment. The experiments were repeated at least twice. Two-way ANOVA combined with Tukey's post-hoc multiple comparison test was conducted to compare groups. *p*-value denotation: '****' $p < 0.0001$.

Sup fig. 4: Sorted naïve CD4 T cells upregulate IL-4Rα in presence of TCR stimulation.

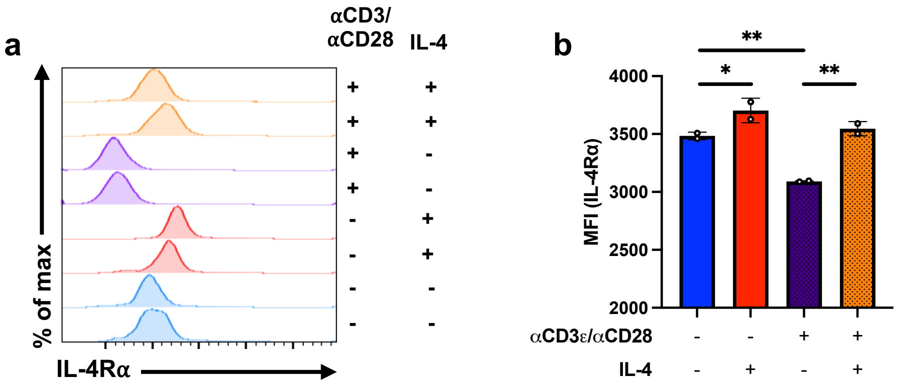

Single cell suspensions from 6–8-week-old female BALB/c mice were sorted by a two-step MACS process, first by negatively selecting CD4 T cells before subsequent positive selection of CD62L^{+} cells. These isolated naïve CD4 T cells were stimulated with 50 ng/mL IL-4 *in vitro* for 24 h prior to flow cytometric analysis. **(a)** Representative histograms show the expression of IL-4Rα on pre-gated CD4 T cells. **(b)** MFI of IL-4Rα was compared between groups. Data are represented as mean + SD. Experiment was completed in duplicates. One-way ANOVA combined with a post-hoc Sidak test was used to compare groups. *p*-value denotation: '*' $p < 0.05$, '**' $p < 0.01$.

Sup fig. 5: Phosphorylation of STAT3 detected under high IL-4 concentrations.

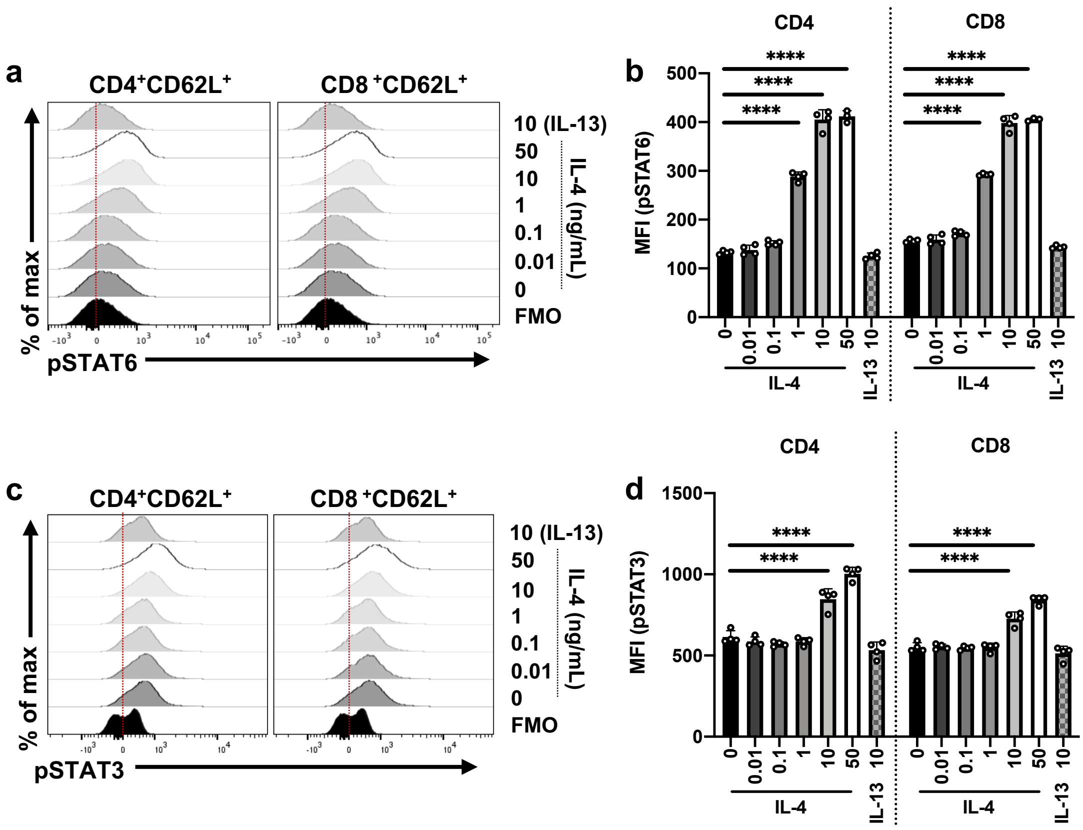

Whole spleen homogenate derived from naïve 6–8-week-old female BALB/c mice was treated with 0.01–50 ng/mL IL-4 or 10 ng/mL IL-13 for 24 h. **(a)** Representative histogram showing pSTAT6 expression among CD62L$^+$ T cells. **(b)** MFI (pSTAT6) with respect to cytokine stimulation among CD62L$^+$ cells. pSTAT3 was also evaluated under different cytokine concentrations **(c–d)**. Data are represented as mean + SD, where dots represent biological replicates from a single experiment. The experiments were repeated at least twice. Two-way ANOVA combined with Tukey's post-hoc multiple comparison test was conducted to compare groups. *p*-value denotation: '***' $p < 0.001$ and '****' $p < 0.0001$.

Sup fig. 6: TGF-β1 expression regulated by IL-4 in a CD62L:CD44 status-dependent manner and IL-6 fails to elicit receptor upregulation.

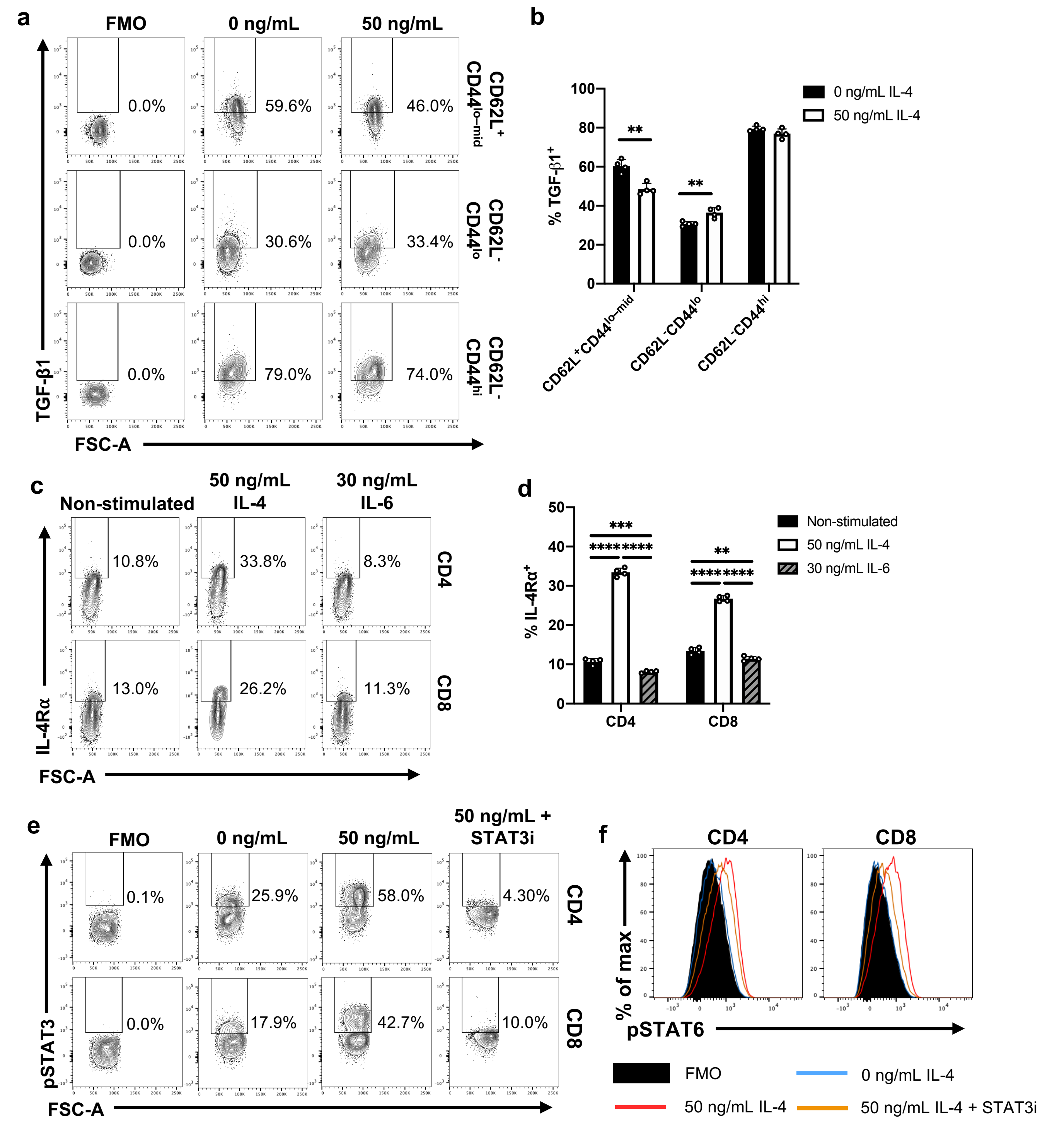

(a) Whole spleen homogenate derived from naïve 6–8-week-old female BALB/c mice were stimulated with 50 ng/mL IL-4 *in vitro* for 24 h. Representative contour plots showing the expression of TGF-β1 with respect to CD62L:CD44 statuses among pre-gated CD4 T cells. **(b)** The percentage of CD4 TGF-β1^{+}. **(c)** Splenic homogenate was alternatively treated with 30 ng/mL IL-6 for 24 h. Representative contour plots. **(d)** The percentage of IL-4Rα^{+} cells. **(e–f)** Splenic homogenate was pre-incubated with STAT3 and/or STAT6 inhibitors prior to IL-4 stimulation. The phosphorylation statuses were subsequently evaluated, with representative contour plots/histograms shown. Data are represented as mean + SD, where dots represent biological replicates from a single experiment. The experiments were repeated twice. Two-way ANOVA combined with Tukey's post-hoc multiple comparison test was conducted to compare groups. *p*-value denotation: '**' $p < 0.01$, '***' $p < 0.001$ and '****' $p < 0.0001$.

Sup fig. 7: Binary analyses of Fluidigm expression profiles and qPCR validation.

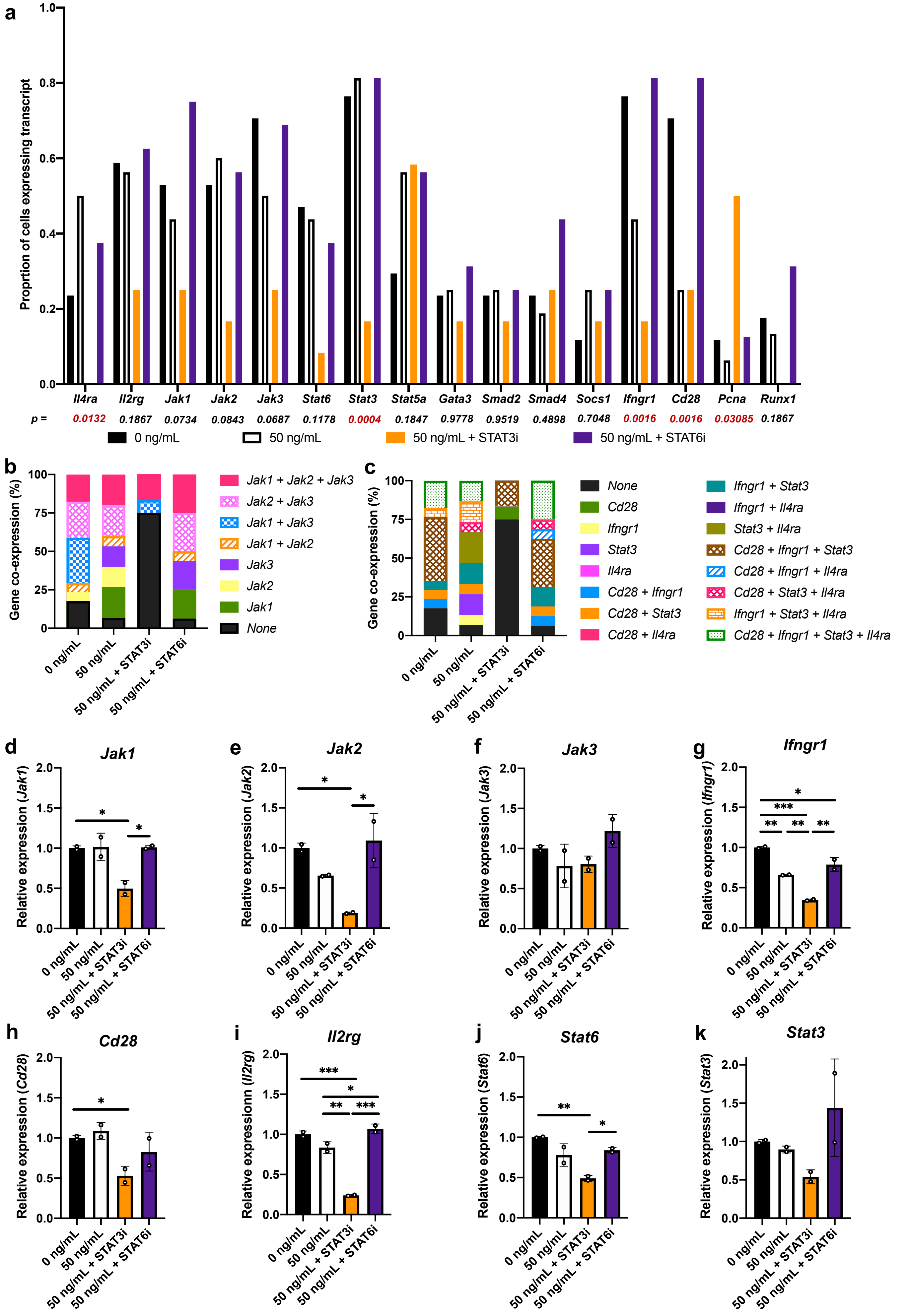

Sup fig 7: Binary analyses of Fluidigm expression profiles and qPCR validation.

Whole spleen homogenate derived from naïve 6–8-week-old female BALB/c mice were pre-incubated with STAT3i or STAT6i *in vitro* for 3 h prior to stimulation with 50 ng/mL IL-4 for 24 h. Fluidigm Biomark 48.48 analyses were performed on single $CD3^{+}CD4^{+}CD8^{-}IL\text{-}4R\alpha^{+}$ cells, evaluating transcript expression of genes-of-interest. **(a)** For genes expressed by at least 15% of all cells, dichotomised expression was evaluated. To evaluate whether the proportion of transcript expression was contingent on treatment, Fisher's exact tests were conducted, where $p < 0.05$ denoted in red. **(b)** Gene co-expression was considered among Jak family members. **(c)** Co-expression profiles of markers-of-interest. The experiment was repeated twice, pooling the data. $n \sim 15$ cells per group. RT-qPCR was performed on 100 sorted $CD3^{+}CD4^{+}CD8^{-}IL\text{-}4R\alpha^{+}$ cells, evaluating transcripts of select markers: **(d)** *Jak1*, **(e)** *Jak2*, **(f)** *Jak3*, **(g)** *Ifnyr1*, **(h)** *Cd28*, **(i)** *yc*, **(j)** *Stat6* and **(k)** *Stat3*. The experiment was completed in duplicates. ANOVA combined with a post-hoc multiple comparison test was conducted to compare groups. *p*-value denotations: '*' $p < 0.05$, '**' $p < 0.01$ and '***' $p < 0.001$.

Sup fig. 8: Clustering analyses.

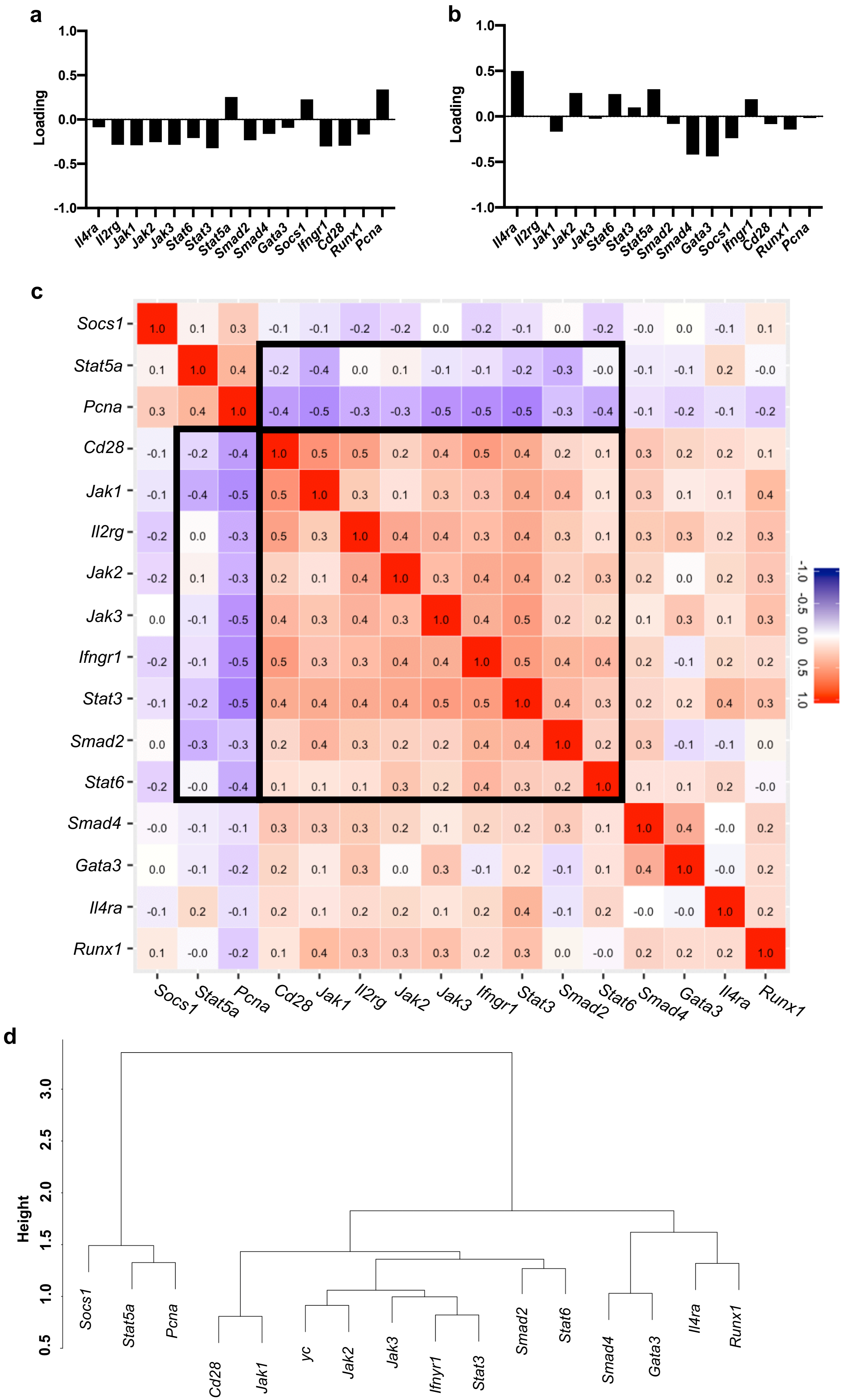

Sup fig 7: Clustering analyses.

Whole spleen homogenate derived from naïve 6–8-week-old female BALB/c mice were pre-incubated with STAT3i or STAT6i *in vitro* for 3 h prior to stimulation with 50 ng/mL IL-4 for 24 h. Fluidigm Biomark 48.48 analyses were performed on single $CD3^{+}CD4^{+}CD8^{-}IL\text{-}4R\alpha^{+}$ cells, evaluating transcript expression of genes-of-interest. For genes expressed by at least 15% of cells, clustering analyses were performed. PCA was conducted, with loadings for both **(a)** PC1 and **(b)** PC2. **(c)** Spearman's rank correlation analysis was performed to evaluate correspondence of genes pairwise. **(d)** Dendrogram is based on the similarity in gene expression.

Sup fig. 9: Flow cytometry gating strategies

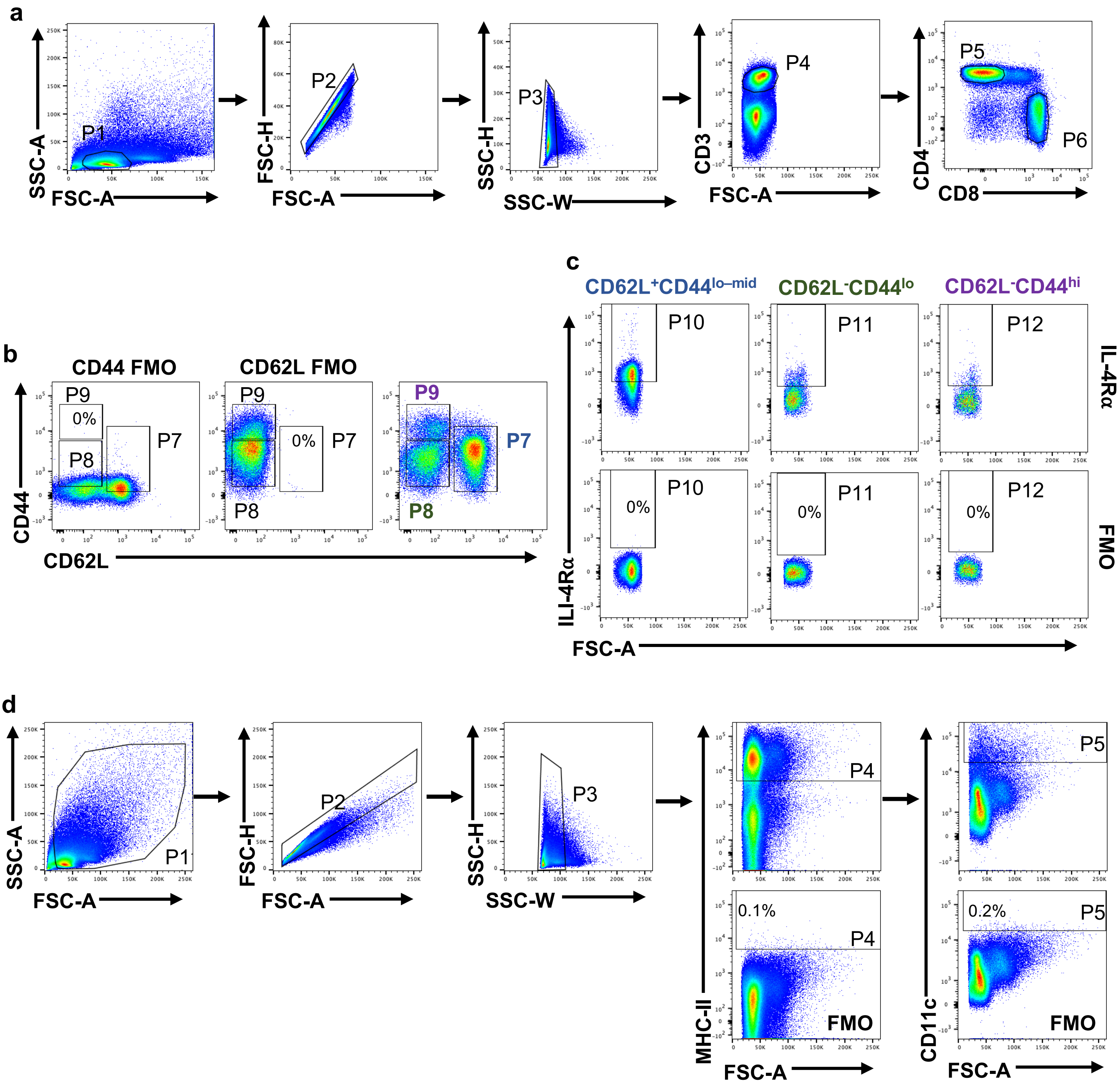

(a) CD4 and CD8 T cells were evaluated by first selecting the lymphocyte population based on FSC-A:SSC-A (P1). This was followed by a two-step doublet discrimination process based on FSC and SSC proportionality (P2; P3). Total T cells were selected according to their CD3 status (P4). CD4 and CD8 T cell subsets were subsequently discerned using the CD4:CD8 bivariate plot (P5; P6). **(b)** Following CD4 and CD8 T cell gating, cells were identified according to their CD62L:CD44 status (the CD4 subset shown here). $CD62L^{+}CD44^{lo–mid}$ (P7), $CD62L^{-}CD44^{lo}$ (P8) and $CD62L^{-}CD44^{hi}$ (P9) according to the respective CD62L and CD44 FMOs. **(c)** Different markers (e.g. IL-4Rα) were subsequently evaluated, according to the relevant FMOs (P10–12). **(d)** APCs were similarly evaluated by first discriminating on the bases of size and granularity (P1), prior to doublet discrimination (P2, P3). $MHC\text{-}II^{+}$ (P4) followed by $CD11c^{hi}$ (P5) events were selected according to their respective FMOs.

Sup fig. 10: ImageStream single-colour controls and gating strategy

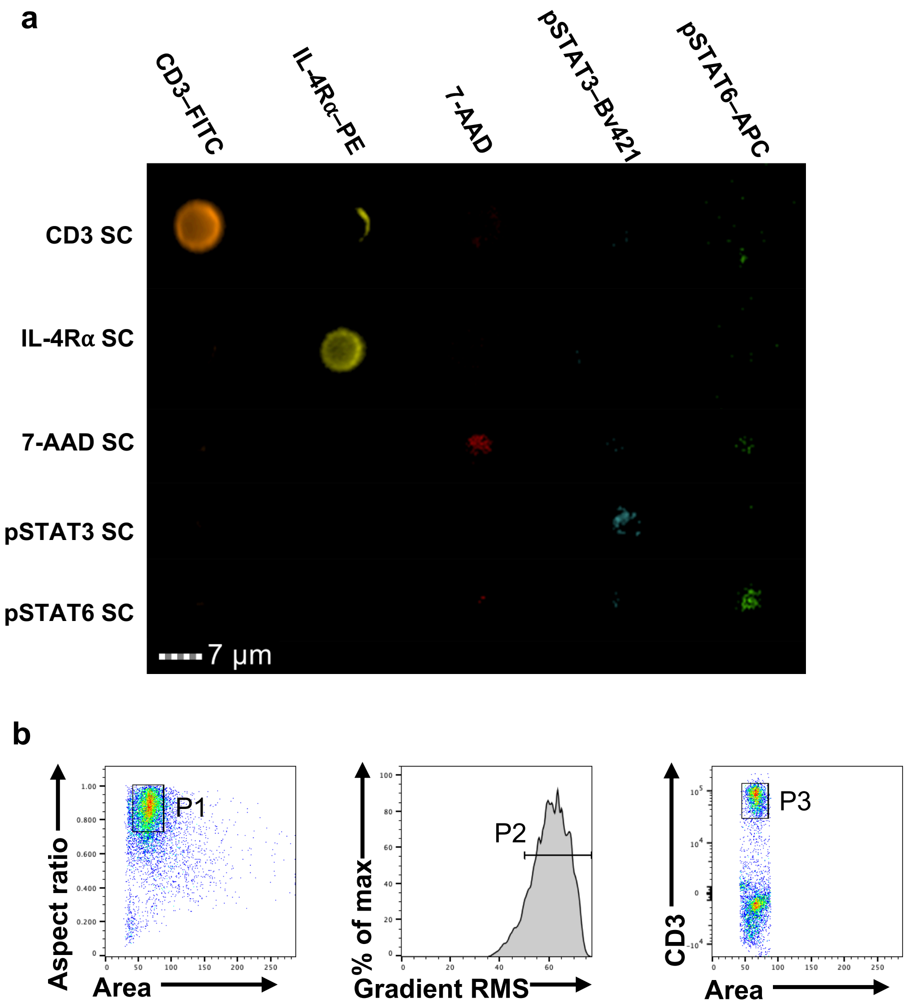

Whole spleen homogenate harvested from naïve WT BALB/c mice were stimulated *in vitro* with 50 ng/mL IL-4 for 24 h prior to ImageStream analysis. **(A)** Single-colour controls were evaluated to set the compensation matrix and to consider spectral overlap. Note that cells were fixed, allowing 7-AAD to be used as a nuclear stain (and is, therefore, not indicative of viability). **(B)** Events were first discriminated based on the area (indicative of event size, as derived from the brightfield channel) and aspect ratio (associated with event circularity and thus can be used to exclude doublets) (P1). High event/flow rate results in cells going out of focus; to ensure that only in-focus events are included in the analysis, gradient RMS was used (P2). Finally, total T cells were selected based on event CD3 status (P3)

Sup table. 1: Assay probes.

Gene symbol	Encoded protein	Assay ID	Amplicon length (bp)	Reference sequence
Il4	IL-4	Mm00445259_m1	79	NM_021283.2
Il13	IL-13	Mm00434204_m1	56	NM_008255.3
Il2	IL-2	Mm_00434256_m1	82	NM_032392.1
Il6	IL-6	Mm_00446190_m1	78	NM_031168.1
Il10	IL-10	Mm01288386_m1	136	NM_010548.2
Il17a	IL-17A	Mm00434214_m1	97	NM_008359.2
Il18	IL-18	Mm00434226_m1	141	NM_008360.1
Tgfb1	Transforming growth factor beta 1	Mm01178820_m1	59	NM_011577.2
Ifng	Interferon gamma	Mm01168134_m1	100	NM_008337.3
Il4ra	IL-4 receptor alpha	Mm0275139_m1	61	NM_001008700.3
Il2rg	γ common chain	Mm0044285_m1	65	AK143669.1
Il13ra1	IL-13 receptor alpha 1	Mm01302068_m1	103	AK080147.1
Il13ra2	IL-13 receptor alpha 2	Mm00515166_m1	105	AK089687.2
Tgfbr1	Transforming growth factor beta receptor 1	Mm00436964_m1	85	NM_009379.2
Il17ra	IL-17 receptor A	Mm00434214_m1	97	NM_008359.2
Il17rb	IL-17 receptor B	Mm00444709_m1	90	NM_019583.3
Il17rc	IL-17 receptor C	Mm00506606_m1	58	NM_598920.3; NM_178942.1
Ifngr1	Interferon-gamma receptor 1	Mm00599890_m1	85	NM_010511.2
Stat6	STAT6	Mm01160477_m1	76	NM_009284.2
Stat3	STAT3	Mm01219775_m1	75	NM_011486.4; NM_213659.2; NM_213660.2
Stat1	STAT1	Mm01257286_m1	75	NM_009283.4; NM_001205314.1; NM_001205313.1
Stat5a	STAT5a	Mm03053818_s1	59	NM001164062.1; NM_011488.3
Socs1	Suppressor of cytokine signalling (SOCS)1	Mm00782550_s1	88	NM001271603.1; NM_009896.2
Socs3	SOCS3	Mm00545913_s1	76	NM_007707.3
Irs1	Insulin receptor substrate 1	Mm01278327_m1	69	NM_010570.4
Irs2	Insulin receptor substrate 2	Mm03038438_m1	63	NM_001081212.1
Jak1	Janus kinase 1	Mm00600614_m1	72	NM_146145.2
Jak2	Janus kinase 2	Mm01208489_m1	62	NM_001048177.2; NM_008413.3
Jak3	Janus kinase 3	Mm00439973_m1	66	NM_001190830.1; NM_010589.6
Smad2	SMAD family member 2	Mm00487530_m1	72	NM_001252481.1; NM_010754.5
Smad4	SMAD family member 4	Mm03023996_m1	71	NM_008540.2
Cd4	Helper T cell marker	Mm00442754_m1	78	NM_013488.2
Cd8a	Cytotoxic T cell marker	Mm01182107_g1	68	NM_001081110.2; NM_009857.1
Cd28	T cell co-stimulatory molecule	Mm01253994_m1	98	NM_001081110.2; NM_009857.1
Ctla4	Cytotoxic T-lymphocyte-associated protein 4	Mm00486849_m1	71	NM_001281976.1; NM_009843.3
Gata3	GATA3	Mm00484683_m1	57	NM_008091.3
Rorc	RAR-related orphan receptor gamma	Mm00484683_m1	54	NM_001293734.1
Runx1	Runt-related transcription factor 1	Mm01213404_m1	81	NM_001111021.2; NM_001111022.2; NM_001111023.2; NM_009821.3
Pcna	Proliferating cell nuclear antigen (PCNA)	Mm05873628_g1	72	NM_011045.2
c-Maf	c-Maf	Mm02581355_s1	154	NM_001020748.2
L32	60S Ribosomal protein	Mm02524691_s1	84	NM_018754.2
Eef2	Eukaryote elongation factor 2	Mm01171435_gH	74	NM_007907.2
